# UHPLC-HRMS-based Multiomics to Explore the Potential Mechanisms and Biomarkers for Colorectal Cancer

**DOI:** 10.1186/s12885-024-12321-7

**Published:** 2024-05-27

**Authors:** Xuancheng Wang, Xuan Guan, Ying Tong, Yunxiao Liang, Zongsheng Huang, Mingsen Wen, Jichu Luo, Hongwei Chen, Shanyi Yang, Zhiyong She, Zhijuan Wei, Yun Zhou, Yali Qi, Pingchuan Zhu, Yanying Nong, Qisong Zhang

**Affiliations:** 1 Guangxi Key Laboratory of Special Biomedicine, School of Medicine, Guangxi University, Nanning, Guangxi 530004 PR China; 2 Department of Academic Affairs, The First Affiliated Hospital of Guangxi Medical University, Nanning, Guangxi 530021 PR China; 3 Department of Gastroenterology, People’s Hospital of Guangxi Zhuang Autonomous Region, Nanning, Guangxi 530021 PR China; 4 Center for Instrumental Analysis, Guangxi University, Nanning, Guangxi 530004 PR China; 5 State Key Laboratory for Conservation and Utilization of Subtropical Agro-Bioresources, Guangxi University, Nanning, Guangxi 530004 PR China

**Keywords:** Colorectal cancer, Lipidomics, Metabolomics, UHPLC-HRMS, Biomarkers

## Abstract

**Background:**

Understanding the metabolic changes in colorectal cancer (CRC) and exploring potential diagnostic biomarkers is crucial for elucidating its pathogenesis and reducing mortality. Cancer cells are typically derived from cancer tissues and can be easily obtained and cultured. Systematic studies on CRC cells at different stages are still lacking. Additionally, there is a need to validate our previous findings from human serum.

**Methods:**

Ultrahigh-performance liquid chromatography tandem high-resolution mass spectrometry (UHPLC-HRMS)-based metabolomics and lipidomics were employed to comprehensively measure metabolites and lipids in CRC cells at four different stages and serum samples from normal control (NR) and CRC subjects. Univariate and multivariate statistical analyses were applied to select the differential metabolites and lipids between groups. Biomarkers with good diagnostic efficacy for CRC that existed in both cells and serum were screened by the receiver operating characteristic curve (ROC) analysis. Furthermore, potential biomarkers were validated using metabolite standards.

**Results:**

Metabolite and lipid profiles differed significantly among CRC cells at stages A, B, C, and D. Dysregulation of glycerophospholipid (GPL), fatty acid (FA), and amino acid (AA) metabolism played a crucial role in the CRC progression, particularly GPL metabolism dominated by phosphatidylcholine (PC). A total of 46 differential metabolites and 29 differential lipids common to the four stages of CRC cells were discovered. Eight metabolites showed the same trends in CRC cells and serum from CRC patients compared to the control groups. Among them, palmitoylcarnitine and sphingosine could serve as potential biomarkers with the values of area under the curve (AUC) more than 0.80 in the serum and cells. Their panel exhibited excellent performance in discriminating CRC cells at different stages from normal cells (AUC = 1.00).

**Conclusions:**

To our knowledge, this is the first research to attempt to validate the results of metabolism studies of serum from CRC patients using cell models. The metabolic disorders of PC, FA, and AA were closely related to the tumorigenesis of CRC, with PC being the more critical factor. The panel composed of palmitoylcarnitine and sphingosine may act as a potential biomarker for the diagnosis of CRC, aiding in its prevention.

**Supplementary Information:**

The online version contains supplementary material available at 10.1186/s12885-024-12321-7.

## Background

Colorectal cancer (CRC), a malignant tumor of the colon or rectum, is the third leading cause of death in both men and women worldwide [1]. Over decades, the prevalence of CRC has increased among individuals less than 50 years of age due to heavy alcohol consumption, obesity, physical inactivity, and unhealthy diet [2, 3]. The survival rate of patients with CRC depends on the stage of the disease at the time of diagnosis, with 92% survival rate for stage I but 10% survival rate for stage IV [4]. Thus, early diagnosis of CRC is particularly important for patients’ survival. However, early detection of CRC is difficult due to the latency of precancerous lesions and low detection rates [5]. Colonoscopy is widely considered the “gold standard” for CRC screening and has been shown to reduce CRC mortality, but it can be costly and invasive [6, 7]. Comparatively, detection based on minimally invasive or non-invasive diagnostic biomarkers offers a simplified method that can improve patients’ compliance [8]. Currently, only some of the reported biomarkers are routinely used in clinical settings [9]. Therefore, it is essential to integrate the metabolic molecular characteristics of CRC at different stages to understand its pathogenesis and provide new evidence for the discovery and clinical application of biomarkers for early diagnosis.

Currently, the exploration of diagnostic biomarkers of CRC mainly focuses on analyses of tissues [10], blood [11], or urine [12]. However, research on tissue-based biomarkers is limited due to invasiveness. Additionally, biomarkers in biofluids may not fully reflect the pathological changes in cancers, which can decrease diagnostic accuracy [13]. Compared to tissues and biofluids, cancer cell models may be more appropriate subjects for study. They are typically derived from cancer tissues, are of good uniformity and accessibility, and can be easily cultured [14]. Furthermore, the pathological process observed in cell culture may reflect the events occurring in cancer tissues. Hence, CRC cells are of significant research value. Accurate staging of CRC is crucial for its diagnosis and treatment. The stage-specific metabolic features of CRC cells have been previously investigated [15]. However, the metabolic profiles at different stages of CRC are still incomplete, and the pathogenesis of CRC at the molecular level remains unclear.

Omics technology, specifically metabolomics, has recently presented a unique advantage in exploring metabolic profiles and discovering novel diagnostic disease biomarkers from numerous biomolecules [16]. Metabolomics is a comprehensive analysis of all metabolites in cells, organs, tissues, or biofluids [17]. These metabolites are related to the metabolic signature and molecular mechanism of disease progression. Lipidomics is a branch of metabolomics, which is defined as the large-scale identification, characterization, and quantification of the structure and function of lipids [18]. Besides, lipidomics can unravel disease-related or disease-specific impairment of lipid homeostasis [19]. In our previous studies on the serum levels of patients with CRC, we have applied a multi-omics analysis strategy combining metabolomics and lipidomics [20, 21]. The results showed that lipids, specifically phosphatidylcholine (PC), play a crucial role in the development of CRC [20, 21]. Therefore, it is worth applying this strategy to investigate CRC cells at different stages to confirm our previous findings and explore the potential mechanisms and biomarkers of CRC.

Thus, we used UHPLC-HRMS-based untargeted metabolomics and lipidomics to construct relatively comprehensive metabolite and lipid profiles of CRC cells at different stages. The common differential metabolites and lipids of CRC cells at different stages were identified by systematically comparing the metabolite and lipid profiles using univariate and multivariate analyses. The diagnostic performance for CRC was measured using the receiver operating characteristic curve (ROC) analysis. More importantly, we used cell omics to validate previous findings from omics studies on the serum samples of patients with CRC. Differential metabolites and lipids with similar changes in CRC cells and human serum samples were selected. Potential biomarkers with good performance for early diagnosis of CRC were explored and validated. This study aimed to provide a more comprehensive understanding of metabolic changes in CRC cells at different stages and explore potential biomarkers for diagnosing CRC. Our findings offer new insights into CRC progression and help early screening and diagnosis of CRC.

## Methods

### Required materials

Normal colon cells (CCD841CoN) and four human-derived CRC cell lines (SW1116, LS180, LOVO, and HCT116 represent stages A, B, C, and D based on Dukes’ classification criteria, respectively) were procured from ATCC, USA repository. Dulbecco’s Modified Eagle Medium (DMEM, high glucose), fetal bovine serum (FBS), phosphate-buffered saline (PBS), and penicillin-streptomycin were obtained from GIBCO (Grand Island, NY, USA). Trypsin-EDTA solution was purchased from Beijing Solarbio Science & Technology Co., Ltd. Liquid chromatography grade methanol, dichloromethane, isopropanol, acetonitrile, formic acid, and ammonium formate were purchased from Merck & Co. (Billerica, MA, USA). A Millipore Milli-Q system was used to prepare the ultrapure water (Billerica, MA, USA). Palmitoylcarnitine and sphingosine standards were purchased from Sigma-Aldrich Shanghai Trading Co. Ltd. (Shanghai, China).

### Cell culture and maintenance

All CRC cells were routinely cultured in DMEM high-glucose culture supplemented with 10% FBS and 1% penicillin-streptomycin and maintained in a humidified CO_2_ incubator with 5% CO_2_ at 37 °C. After reaching 80–90% confluence, the culture fluid was discarded. Then, 1 mL trypsin-EDTA solution was added and all cells were subcultured every two days at a ratio of 1:2. Cells in the logarithmic phase were collected for the following experiments.

### Sample preparation

Logarithmic phase cells were washed with pre-cooled 0.9% saline 5–6 times and transferred to 1.5 mL Eppendorf tube following centrifugation at 1000 rpm/min for 5 min at 4 °C. The supernatant was collected to extract metabolites and lipids of cancer cells.

### Cell sample preparation for metabolome assay

We prepared samples following our previous study with some modifications [21]. 400 µL of precooling acetonitrile was added to the above-mentioned supernatant. After being vortexed for 5 min, the mixture was ultrasonicated twice to lyse cells, 15 s each time. Then, the mixture was ultrasonicated in an ice bath for 5 min. The suspension was centrifuged at 14,000 rpm/min at 4 °C for 10 min, and 350 µL of supernatant was dried in vacuum at room temperature. The dried samples were redissolved with 100 µL acetonitrile-water (1:1, v/v) solution. Finally, the samples were centrifuged at 14,000 rpm/min at 4 °C for 10 min, and 90 µL of supernatant was absorbed for metabolomic analysis. Quality control (QC) samples were prepared by mixing an equal amount of each sample to investigate the repeatability and stability of the analytical system.

### Cell sample preparation for lipidome assay

To obtain better efficiency in lipids extraction, we used a solvent mixture, including dichloromethane and methanol, because it is suitable for extracting hydrophobic metabolites based on their physicochemical property [16]. 400 µL of precooling dichloromethane-methanol (3:1, v/v) solution was mixed with the prepared supernatant of logarithmic phase cells. The mixture was vortexed for 5 min, placed in the ice bath for 10 min, and ultrasonicated twice, 15 s each, to lyse cells. Next, the mixture was ultrasonicated in an ice bath for 5 min and centrifuged at 14,000 rpm/min at 4 °C for 10 min. Thereafter, 300 µL of lower dichloromethane solution was dried in a vacuum at room temperature. The dried samples were redissolved with 300 µL acetonitrile-isopropanol (1:1, v/v) solution, vortexed for 2 min, and ultrasonicated in an ice bath for 5 min. After being vortexed for 1 min, the mixture was centrifuged at 14,000 rpm/min at 4 °C for 10 min. Next, 100 µL of supernatant was used for lipidomic analysis. The preparation method of QC samples was the same as above.

### UHPLC-HRMS analysis on CRC cells

Untargeted metabolomic and lipidomic analyses were conducted using a Dionex Ultimate 3000 liquid chromatography system (Sunnyvale, CA, USA) (SN: 7254012). Tandem mass spectrometry was performed with a Thermo Fisher Q Exactive Orbitrap MS (Waltham, MA, USA) (SN: SN02386L) equipped with a Waters Acquity UPLC HSS T3 column (1.8 μm, 2.1 × 100 mm) (Milford, MA, USA). The analysis was conducted following our previous study with some modifications [21, 22]. The detailed LC and MS conditions were provided in Tables S1- S3 (see Additional file 1). All data were obtained using Thermo Scientific Xcalibur 3.1 software (Waltham, MA, USA).

### Multiomics study of patients with CRC

Data for UHPLC-HRMS-based metabolomics and lipidomics were obtained from the serum samples of patients with CRC in our previous study [21]. The study was approved by the Ethics Committee of the People’s Hospital of Guangxi Zhuang Autonomous Region (number KY-DZX-202008), and all participants signed the informed consent forms. In total, 161 subjects (79 normal control (NR) and 82 patients with CRC) were recruited for serum metabolomics analysis, and 100 subjects (50 NR and patients with 50 CRC) were enrolled for serum lipidomics analysis. Their baseline characteristics were provided in Table S4 (see Additional file 1). Inclusion criteria for patients with CRC were as follows: [1] Patients with pathological confirmation of CRC; [2] Patients without any metabolic diseases, such as diabetes, kidney, liver diseases, hematologic diseases, or other cancers; and [3] Patients who did not undergo surgery, chemotherapy, or radiotherapy before enrolment. The inclusion criteria for NR subjects were as follows: [1] absence of CRC or other cancers; [2] no history of CRC in the family; [3] no metabolic diseases, such as hypertension, hyperlipidemia, or diabetes; [4] no lipid-altering medications in the last month; and [5] no intestinal tumors of any kind in colonoscopy. Using coagulation tubes, serum samples of the study subjects were collected after 8-h of fasting.

### Data processing and visualization

Both metabolomics and lipidomics data were imported to Compound Discoverer^TM^3.1 (Thermo Scientific, Fremont, CA, USA). Three-dimensional data including retention time (RT), *m/z*, and peak intensity were extracted. Normalization of peak intensity was conducted using QC samples to find differential metabolites and lipids between the two groups. Inter-group difference analysis was performed using the Mann-Whitney U test or *t*-test. *P* < 0.05 was considered statistically significant. A potential metabolite list was identified based on Thermo mzCloud and HMDB (https://hmdb.ca/). A list of potential lipids was identified according to Thermo mzCloud and mzVault with LipidBlast database. Metabolites and lipids with match scores > 70 were considered valid for identification and annotation. Meanwhile, the structures of identified metabolites and lipids were determined by precursor and fragment mass error (10 ppm) and RT tolerance (0.2 min).

Multivariate analysis was conducted using SIMCA-P 14.1 (Umetrics, UMEA, Sweden). Principal component analysis (PCA) and orthogonal partial least squares discriminant analysis (OPLS-DA) were used to explore differences in metabolite and lipid profiles between groups. A 200 times permutation test was conducted to prevent overfitting. The values of R2 (explanatory parameter) and Q2 (predictive parameter) were used to assess the predictability and plausibility of the OPLS-DA model. Fold change (FC) > 2.0 or < 0.5 and *P* < 0.05 were applied to the cell omics study to select differential metabolites and lipids between the two groups. The selection criteria for omics analysis of human serum samples were FC > 1.5 or < 0.67 and *P* < 0.05. ROC analysis was perfromed to explore biomarkers of CRC and measure their diagnostic performance. ROC analysis was conducted using MetaboAnalyst 5.0 (https://www.metaboanalyst.ca/) and GraphPad 8.0.1.

## Results

### Overall differential metabolite and lipid profiles between normal colon cells and CRC cells

**Fig. 1:**
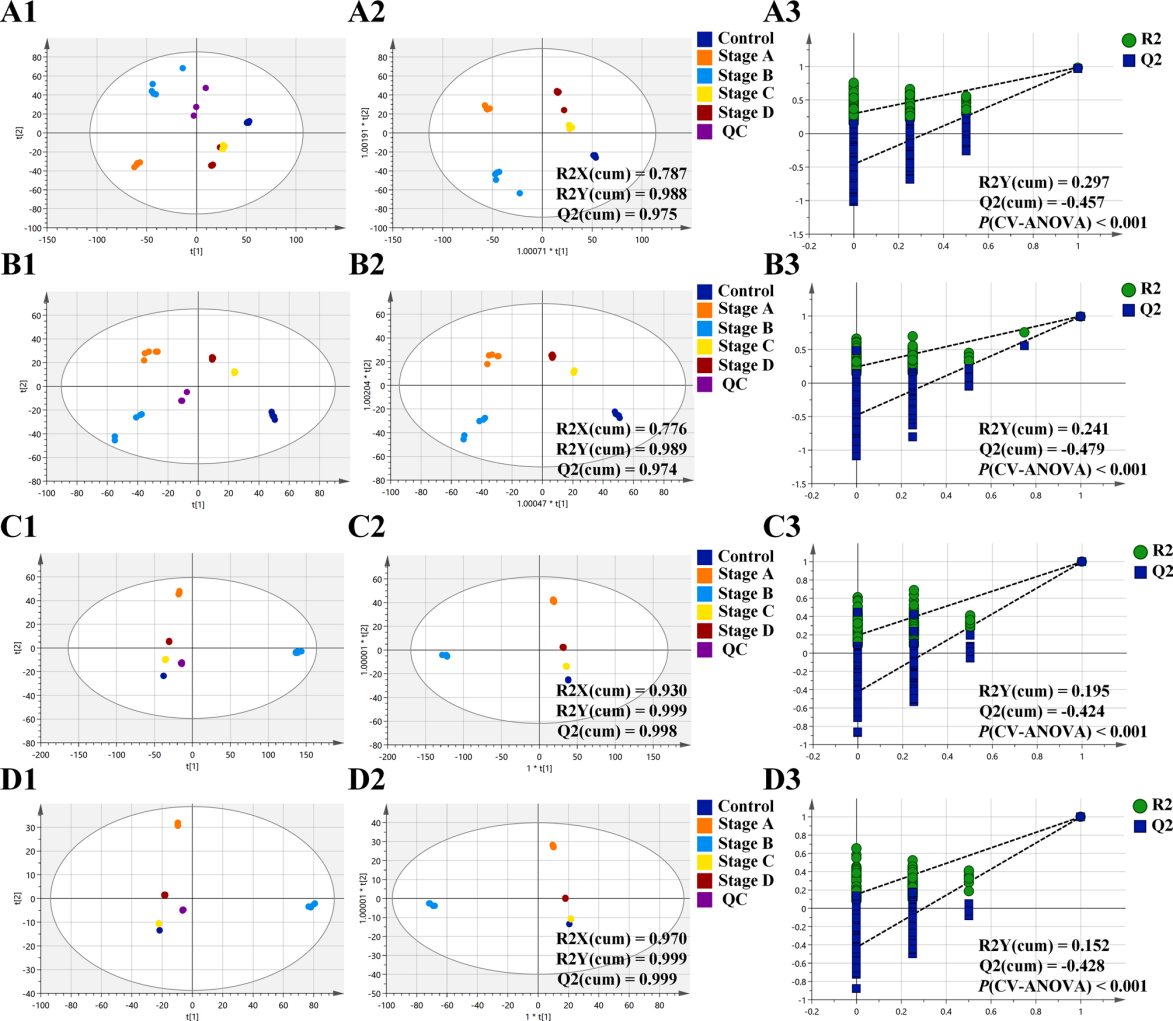
Multivariate statistical analysis of metabolite and lipid profiles among five groups in both ESI modes (*n* = 5 for each group). Principal component analysis (PCA) score plot, orthogonal partial least squares discriminant analysis (OPLS-DA) score plot and permutation test among five groups based on metabolomics in ESI + mode (**A1**-**A3**); PCA score plot, OPLS-DA score plot and permutation test among five groups based on metabolomics in ESI- mode (**B1-B3**); PCA score plot, OPLS-DA score plot and permutation test among five groups based on lipidomics in ESI + mode (**C1-C3**); PCA score plot, OPLS-DA score plot and permutation test among five groups based on lipidomics in ESI- mode (**D1-D3**). Abbreviations: ESI, electrospray ionization; QC, quality control

The PCA model was first built to evaluate the natural clustering of samples and to identify outliers. PCA score plots showed that QC samples were closely clustered in both ESI modes in metabolomic and lipidomic analysis. This finding indicated that the analysis system and detection methods presented good robustness and reproducibility during the batch analysis of samples. In addition, clear separation between the control cell group and the CRC cell group was observed in the PCA analysis, confirming their evident differences in metabolite and lipid profiles (Fig. 1A1, B1, C1, and D1). Next, the OPLS-DA model was established to describe inter-group differences. The results showed that all five groups were well discriminated (Fig. 1A2, B2, C2, and D2). Meanwhile, 200 times permutation tests were conducted to assess the reliability and applicability of the OPLS-DA model. The results indicated that the OPLS-DA model was rational and not overfitted for data analysis of metabolomics and lipidomics (Fig. 1A3, B3, C3, and D3).

### Exploration of metabolites and lipids distinguishing CRC cells from normal colon cells

**Fig. 2:**
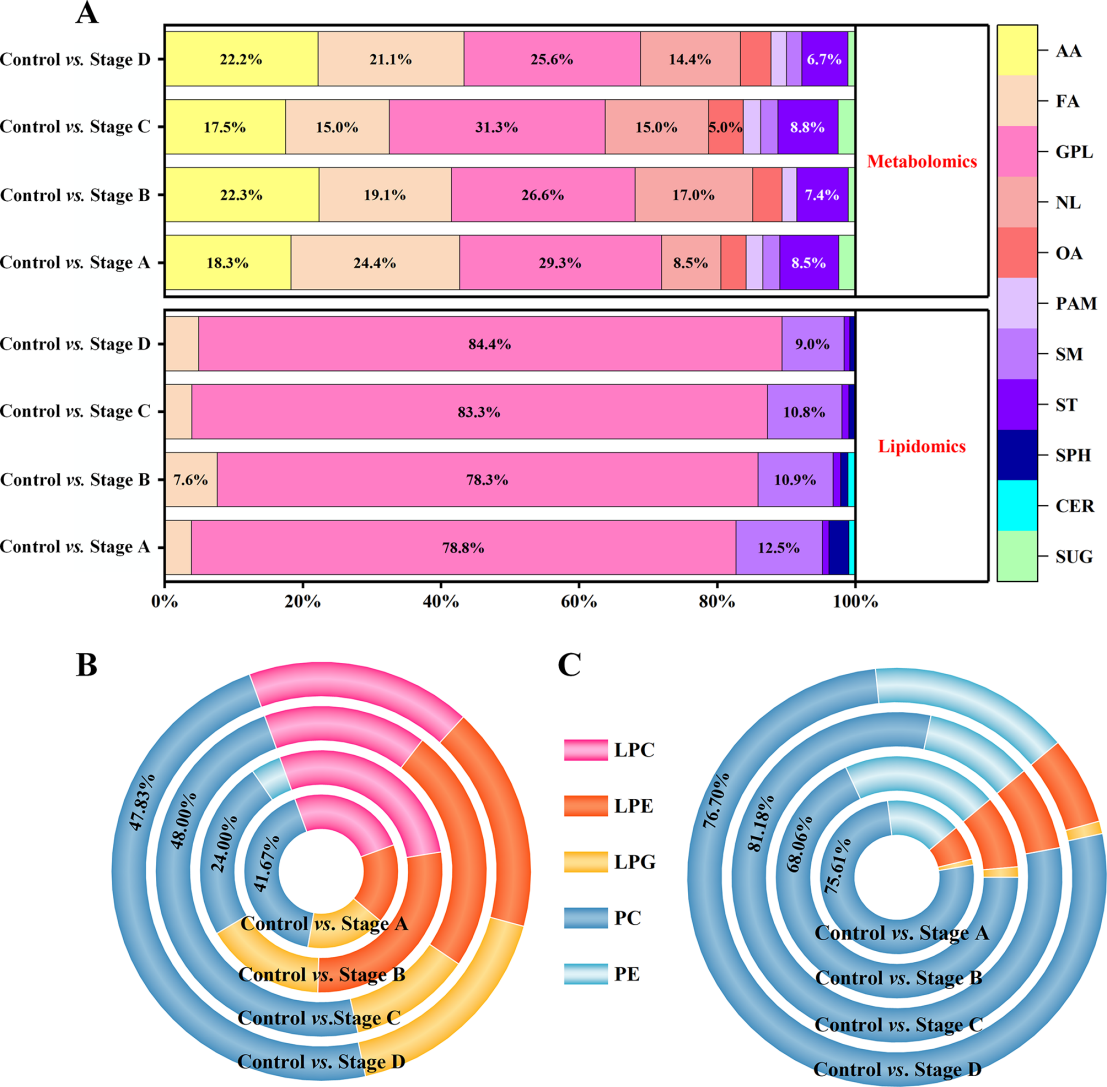
Distribution profile of differential metabolites and lipids at four stages of CRC cells. The constituent ratio of differential metabolites and lipids in the comparison of Control *vs.* Stage A, Control *vs.* Stage B, Control *vs.* Stage C, and Control *vs.* Stage D (**A**); The compositional proportions of glycerophospholipids of CRC cells at four stages by metabolomics (**B**); The compositional proportions of glycerophospholipids of CRC cells at four stages by lipidomics (**C**). Abbreviations: CRC, colorectal cancer; AA, amino acid; FA, fatty acid; GPL, glycerophospholipid; NL, nucleotide; OA, organic acid; PAM, polyamine; SM, sphingomyelin; ST, steroid; SPH, sphingosine; CER, ceramide; SUG, sugar. LPC, lysophosphatidylcholine; LPE, lysophosphatidylethanolamine; LPG, lysophosphatidylglycerol; PC, phosphatidylcholine; PE, phosphatidylethanolamine

Based on differences in metabolite and lipid profiles, combined with FC > 2.0 or < 0.5 and *P* < 0.05, 82, 94, 80, and 90 differential metabolites were selected between Control and Stage A, Control and Stage B, Control and Stage C, and Control and Stage D, respectively. Differential metabolites mainly included amino acid (AA), fatty acid (FA), glycerophospholipid (GPL), nucleotide (NL), organic acid (OA), polyamine (PAM), sphingomyelin (SM), steroid (ST), and sugar (SUG) (Fig. 2A). Among them, lipids were the main component in CRC cells at different stages, with more than 50% composition rate (Fig. 2A). GPL accounted for the most proportion with more than 25% composition rate in the total differential metabolites of four stages of CRC cells. After GPL, FA and AA had the highest composition rates (Fig. 2A). Furthermore, the percentage of PC was significantly higher in CRC cells (Fig. 2B).

We conducted a clustering heatmap analysis to investigate the distribution and trend of differential metabolites between the two groups. The findings revealed that compared to the control cell group, the levels of most differential metabolites were significantly upregulated in CRC cells at different stages (*P* < 0.05, Figures S2- S5) (see Additional file 1).

Owing to the limitation of the sample preparation and lipid extraction method in metabolomics, we systematically and extensively investigated the dysregulation of lipid metabolism in CRC cells. Similarly, differential lipids in between-group analysis were screened based on the above-mentioned criteria (FC > 2.0 or < 0.5, *P* < 0.05). In addition, 104, 92, 102, and 122 differential lipids were selected by comparing Control and Stage A, Control and Stage B, Control and Stage C, and Control and Stage D, respectively. The lipids distinguishing CRC cells from normal colon cells were grouped into several classes, including FA, GPL, SM, ST, sphingosine (SPH), and ceramide (CER) (Fig. 2A). Interestingly, similar to metabolomics results, GPL disorders were most evident in the four stages of CRC with a composition ratio of more than 75% (Fig. 2A). Moreover, PC took up the highest ratio of differential GPL (more than 65%), implying that PC metabolism was most obviously dysregulated in CRC (Fig. 2C). Clustering of differential lipids unveiled that most PC were notably upregulated in CRC cell groups compared to the control cell group (*P* < 0.05, Figures. S6-S9) (see Additional file 1).

### Searching for differential metabolites and lipids common in different stages of CRC

**Fig. 3:**
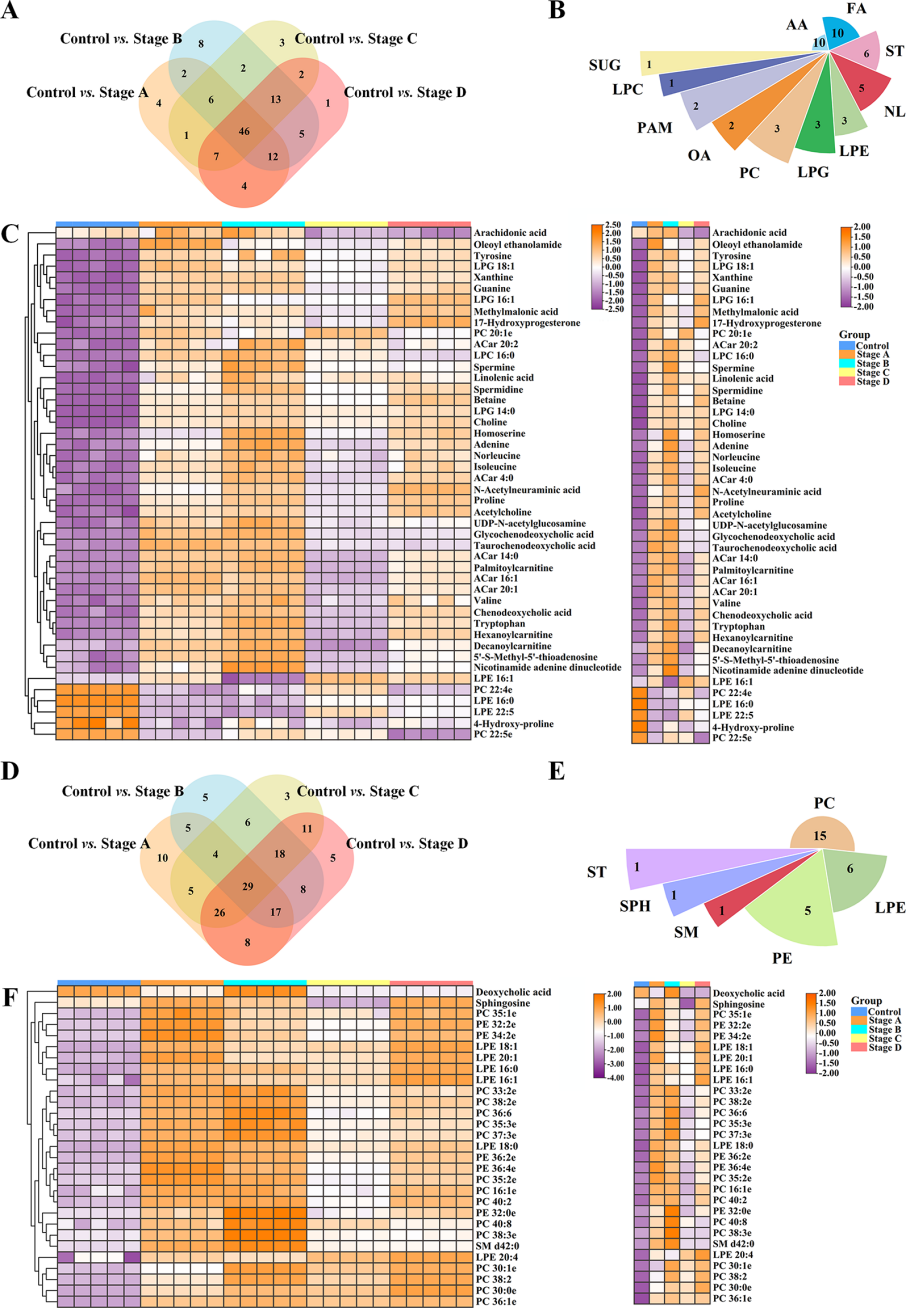
Exploration of differential metabolites and lipids common to the CRC cells in different stages. Venn diagram analysis for the differential metabolites and lipids in the comparison between groups based on the results of metabolomics and lipidomics, respectively (**A, D**); Nightingale rose diagram composition of 46 shared differential metabolites and 29 shared differential lipids of four different stages of CRC cells, respectively (**B, E**); Heatmaps and corresponding average level distribution for shared differential metabolites and lipids in CRC cells at four stages by metabolomics and lipidomics analysis, respectively (**C, F**). Abbreviations: CRC, colorectal cancer; AA, amino acid; FA, fatty acid; ST, steroid; NL, nucleotide; LPE, lysophosphatidylethanolamine; LPG, lysophosphatidylglycerol; PC, phosphatidylcholine; OA, organic acid; PAM, polyamine; LPC, lysophosphatidylcholine; SUG, sugar; PE, phosphatidylethanolamine; SM, sphingomyelin; SPH, sphingosine

Molecular characterization of CRC cells is crucial for diagnosing CRC. The association of differential metabolites and lipids of the four groups was confirmed by Venn diagram analysis. Common differential metabolites and lipids may be potential diagnostic biomarkers for CRC. Therefore, their diagnostic performance was evaluated, and their area under the curve (AUC) values are listed in Tables S7- S8 (see Additional file 1). The results of metabolomics showed a high similarity of differential metabolites among the groups. We identified 46 differential metabolites of CRC cells shared between different stages, including 34 differential metabolites in the ESI + mode and 12 differential metabolites in the ESI- mode (Fig. 3A and Table S7) (see Additional file 1). The composition of the 46 differential metabolites included 10 AA, 10 FA, 6 ST, 5 NL, 3 LPE, 3 LPG, 3 PC, 2 OA, 2 PAM, 1 LPC, and 1 SUG (Fig. 3B). Lipids constituted more than 50% (26/46) of components, suggesting that abnormal lipid metabolism is the most common metabolic abnormality in CRC (Fig. 3B). Particularly, AA and FA occupied the first place with 46 shared metabolites in different stages of CRC. This suggests that the metabolism of AA and FA may be involved in the development of CRC.

In lipidomics, there were 29 shared differential lipids at different stages of CRC, with 12 in the ESI + mode, and 17 in the ESI- mode (Fig. 3D and Table S8) (see Additional file 1). These shared differential lipids included 15 PC, 6 LPE, 5 PE, 1 SM, 1 SPH, and 1 ST (Fig. 3E). PC was also the key dysregulated category, which was consistent with the composition of differential lipids shown in Fig. 2. Cluster analysis showed that compared to normal colon cells, differential metabolites and lipids shared at different stages of CRC were mostly upregulated (*P* < 0.05, Fig. 3C and F). Therefore, the results of lipidomics confirmed that disruption of PC metabolism may be the key determinant in the malignant progression of CRC.

### Evaluating the performance of potential biomarkers for diagnosing CRC

**Fig. 4:**
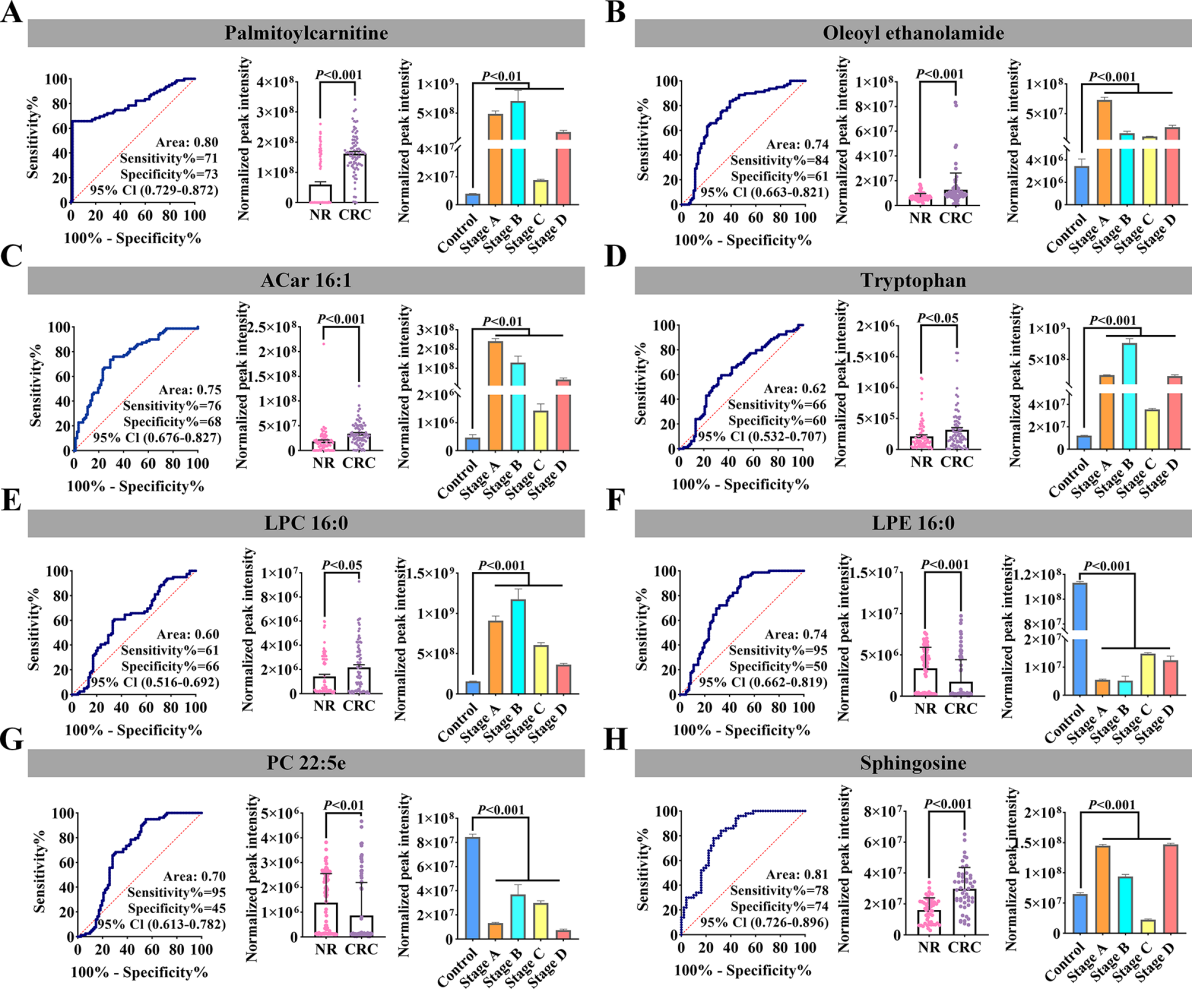
Performance evaluation of eight differential metabolites and lipids with the same change trend in samples of CRC cells and serum samples of CRC patients compared to the control cell group and NR. Data were exhibited with mean ± SD. Abbreviations: NR, normal control; CRC, colorectal cancer

After selecting the common differential metabolites and lipids at different stages of CRC, ROC analysis was applied to evaluate their diagnostic performance for CRC. The AUC values of most differential metabolites and lipids were relatively high (Tables S7- S8) (see Additional file 1). Then, we measured the changing trend of these common differential metabolites and lipids Those with the same change trend in the serum of patients with CRC deserve further research. Finally, eight differential compounds including palmitoylcarnitine, oleoyl ethanolamide, ACar 16:1, tryptophan, LPC 16:0, LPE 16:0, PC 22:5e, and sphingosine were identified (Fig. 4). Among them, LPE 16:0 and PC 22:5e were significantly upregulated in CRC compared to the control group, while the remaining differential metabolites were significantly downregulated (*P* < 0.05, Fig. 4).

ROC analysis was conducted on these eight differential metabolites in the serum of patients with CRC (Fig. 4). Among them, palmitoylcarnitine and sphingosine exhibited good discrimination performance for CRC (AUC > 0.80), with relatively high sensitivity and specificity (more than 0.70). The AUC values of these two compounds for different stages of CRC were almost 1.00 (except for palmitoylcarnitine at stage C of CRC with an AUC value of less than 1.00).

**Fig. 5:**
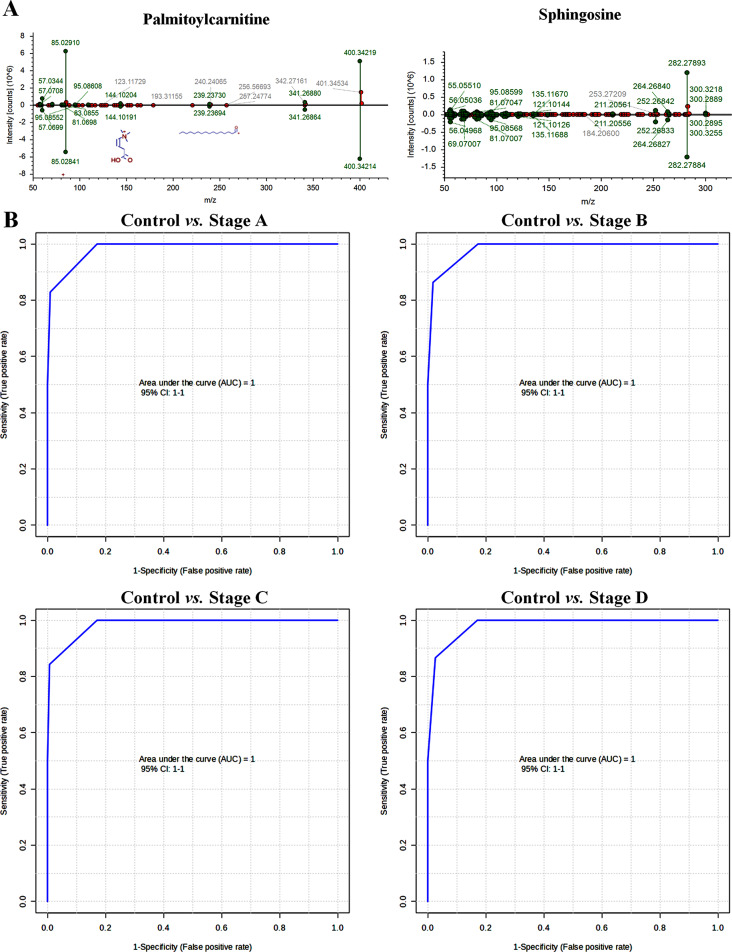
Identification of two differential metabolites with high diagnostic ability for CRC patients and the performance evaluation of their panel in CRC cells with different stages. Abbreviations: CRC, colorectal cancer

To confirm these two differential compounds, their precursor and fragments of metabolite standards were matched with MS information from the database using the same data acquisition method. The results showed that the two metabolite standards matched well with the MS and MS/MS spectra of the database, and the high-resolution mass error was less than 10 ppm (Fig. 5A).

Furthermore, to seek an optimized diagnostic model, multivariate ROC analysis was employed on a biomarker panel consisting of palmitoylcaritine and sphingosine. The panel showed excellent performance in discriminating CRC cells from normal colon cells, with AUC values of 1.00 in all diagnostic models (Fig. 5B). This panel may improve the diagnosis of CRC, therefore, palmitoylcarnitine and sphingosine have great potential as diagnostic biomarkers for CRC.

## Discussion

CRC is a global public health problem with a serious effect on people’s health. Metabolic reprogramming is a significant hallmark of cancer development and progression. Therefore, it is crucial to monitor the levels of metabolites associated with cancer tissue and cancer cells and explore potential biomarkers for accurate diagnosis and treatment of cancer. As tissue biopsy is invasive and relatively difficult, we chose human CRC cell lines as the subject of our study. CRC cell lines were classified based on Dukes’ classification criteria by ATCC and previous studies [15, 23]. The Dukes’ staging system classifies CRC into four groups known as Dukes’ A, B, C, and D groups, which represent stages I, II, III, and IV of CRC. Each stage is described as follows [24] [1]. Stage A represents superficial tumors limited to the mucosa; [2] Stage B represents CRC without lymph node metastases, infiltrating the submucosa, but not infiltrating muscularis propria or infiltrating extra-rectal tissues; [3] Stage C represents CRC with lymph node metastasis [4]. Stage D represents CRC with distant systemic spread or direct invasion of the peritoneum.

By combining metabolomics and lipidomics, we uncovered the metabolic changes at different stages of CRC. Our results were used as a valid supplement to our previous findings from human serum. The results of cell metabolomics showed that GPL, FA, and AA were the top three differential metabolites. Additionally, the results of cell lipidomics indicated that GPL constituted more than 75% of the total composition of all differential lipids. Similar results were obtained after analyzing differential metabolites and lipids shared in different stages of CRC. Thus, the abnormal metabolism of GPL may be the main factor in the development of CRC. Additionally, abnormal metabolism of FA and AA are important features of CRC cells.

**Fig. 6:**
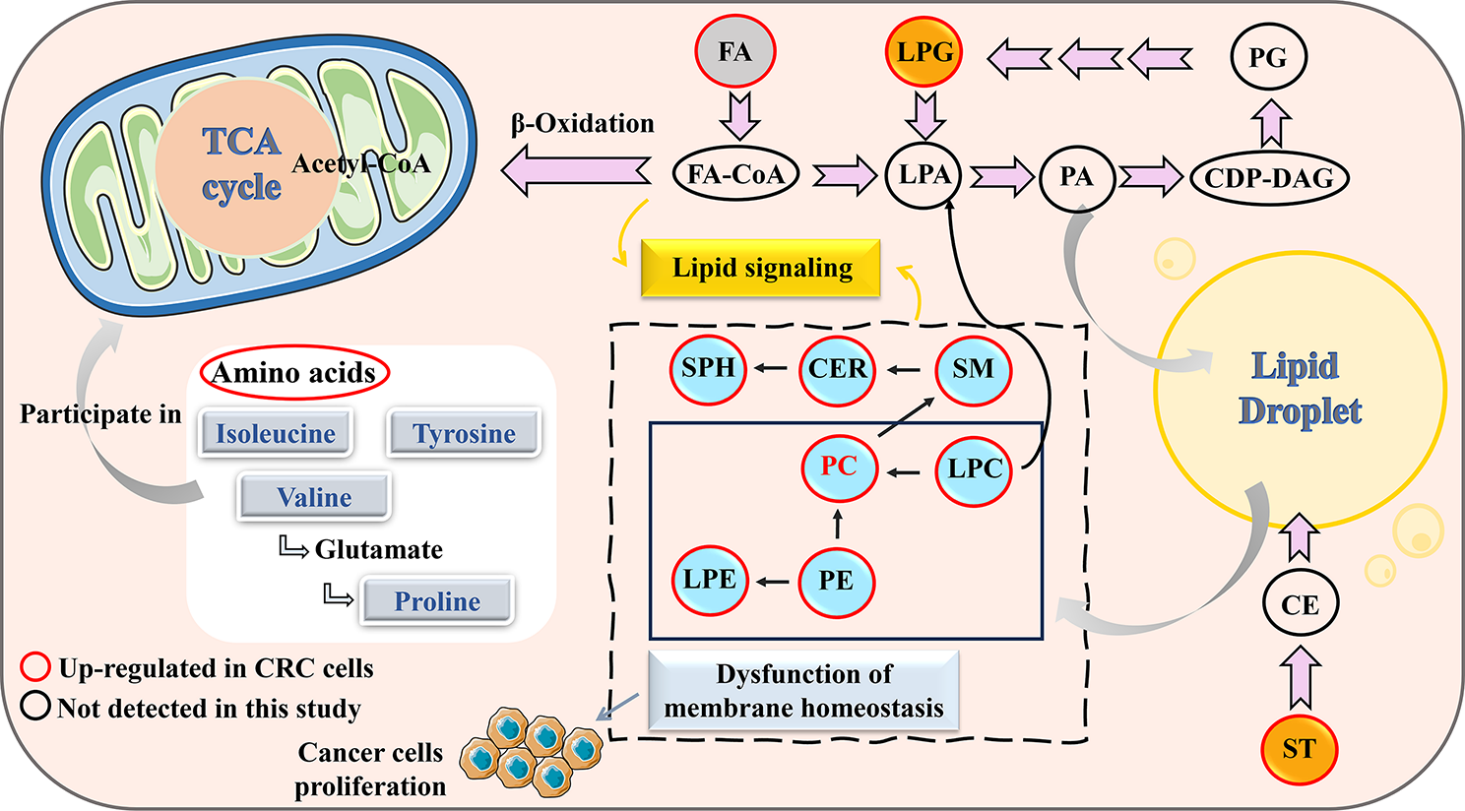
Diagram of potential metabolic mechanism involved in the progression of CRC cells with different stages. Abbreviations: CRC, colorectal cancer; FA, fatty acid; LPA, lysophosphatidic acid; PA, phosphatidic acid; CDP-DAG, cytidinediphosphate-diacylglycerol; PG, phosphatidylglycerol; LPG, lysophosphatidylglycerol; ST, steroid; CE, cholesterol ester; LPE, lysophosphatidylethanolamine; PE, phosphatidylethanolamine; PC, phosphatidylcholine; LPC, lysophosphatidylcholine; SM, sphingomyelin; CER, ceramide; SPH, sphingosine

A metabolic pathways diagram was visualized to help understand the metabolic changes in CRC (Fig. 6). The map depicted that all classes of lipids, particularly phospholipids, were upregulated in CRC cells compared with normal colon cells. Lipids have vital physiological functions. In addition to being the main components of cell membranes, they are involved in the synthesis and regulation of biological macromolecules and serve as second messengers and energy reservoirs [25]. Previous studies indicated that phospholipids levels increase in CRC tissue, possibly due to increased synthesis of plasma membranes in rapidly proliferating cancer cells [26]. Thus, the observed phenomenon in CRC cells is likely caused by the accelerated multiplication of cancer cells. GPL accounted for the highest proportion of differential metabolites and lipids, suggesting that impaired GPL metabolism may be the most important factor in CRC progression. It is clear that PC was the main component in differential GPL composition and differential lipids shared in different stages of CRC cells (Fig. 3E). PC level is generally increased in CRC, and promotes the growth of CRC cells by regulating intracellular signaling pathways [27]. Phosphocholine is a substrate needed for PC synthesis. CKα, which produces phosphocholine from choline, is overexpressed in CRC tissues. In addition, it was observed that increased CKα levels correlated with tumor metastasis [28, 29] LPC is a degradation product of PC catabolism. Studies have shown that LPC can activate macrophages, maintain the M1 phenotype of macrophages, produce IL-12, IL-1β, TNF-α, and IL-6, and other proinflammatory factors, enhance the inflammatory response, and promote the development of CRC [30, 31]. PE can catalyze the synthesis of PC, explaining the increased levels of PC in CRC [32]. Furthermore, PE is enriched in the mitochondria inner membrane. They regulate key mitochondrial functions such as energy production [33]. LPE is the hemolytic product of PE and is involved in intercellular signal transduction through G protein-coupled receptor (GPCR) [34]. Hofmanová et al. reported that LPE level is significantly higher in colon cancer tissues than in normal colon tissues [35].

FA molecules can act as building blocks for newly synthesized membrane phospholipids in highly proliferative CRC cells [36]. Our previous study found that abnormal FA metabolism may be a crucial factor in the malignant transformation of colorectal adenoma [20]. Cells with increased lipid uptake usually present enhanced FA oxidation (FAO) [37]. FA is catabolized via beta-oxidation, which not only fuels cancer cells but also generates the reducing power needed for attenuating oxidative stress during cancer progression [38, 39]. CPT1A on the outer mitochondrial membrane is the rate-limiting enzyme of beta-oxidation, regulating the entrance of FA into the mitochondria. CPT1A is highly expressed in CRC cells, enhances FAO, and promotes the survival and distant metastasis of CRC cells [40]. In our study, the 10 FAs shared in different stages of CRC were mainly involved in linoleic acid metabolism and carnitine metabolism (mainly acetylcarnitine metabolism) (Table S7) (see Additional file 1). Linoleic acid metabolism plays an important role in inflammation [41]. It has been proven that linoleic acid is associated with disease stage, tumor localization, and cancer progression in patients with CRC [42]. Carnitine is a natural-origin AA and essential metabolite in the liver, kidneys, and placenta [43]. It is involved in lipid metabolism and beta-oxidation in humans, which can transport FA into the mitochondria for oxidation and energy production [44].

Most of the acetyl-CoA produced by FAO is used in the TCA cycle, also known as the citric acid cycle. The TCA cycle is the hub of glucose, FA, and AA metabolism. AA are essential nutrients for humans and important energy sources. Remarkable changes in the concentration of AA have been observed in patients with CRC [45]. Furthermore, we found that some AAs were present in CRC cells (Table S7) (see Additional file 1). Tyrosine was confirmed to be a biomarker for early-stage CRC [46]. Proline is known to contribute to tumor cell survival as an AA that is released during cell stress [47]. Isoleucine and valine can be catabolized to acetyl-CoA, which also modulates protein acetylation and promotes tumor growth [48]. To sum up, abnormal metabolism of FA, AA, and particularly GPL, was closely associated with CRC development.

CRC is usually diagnosed at an advanced stage, which lowers cure rates and long-term survival [49]. Therefore, exploration of potential biomarkers for the early diagnosis of CRC is necessary. Among eight differential metabolites with similar trends in CRC cells and patients’ serum samples, palmitoylcarnitine and sphingosine exhibited good diagnostic performance for CRC in ROC analysis. Palmitoylcarnitine is an important endogenous FA metabolite. Previous studies indicated that it can decrease the survival of CRC cells by depleting glutathione [50]. Perturbations in sphingolipid metabolism may contribute to CRC progression [51]. Increased intracellular levels of sphingosine, the major component of sphingolipids, are typically associated with cell cycle arrest and/or cell death [52]. Similarly, potential biomarkers identified in this study were significantly upregulated in CRC (*P* < 0.05, Fig. 4). Interestingly, the AUC value of palmitoylcarnitine was 0.64 for stage C CRC, while the AUC value of the panel, including palmitoylcarnitine and sphingosine, was 1.00 for distinguishing normal colon cells from CRC cells at different stages (Fig. 5B). This provided additional evidence for the use of palmitoylcarnitine, sphingosine, and their panel as biomarkers for diagnosing CRC.

In summary, the integrated analysis of metabolomics and lipidomics systematically revealed the metabolite and lipid profiles of CRC cells at stages A, B, C, and D. PC metabolism may be the most critical factor in the pathogenesis of CRC. Besides, FA and AA metabolism were also closely correlated with CRC progression. Palmitoylcarnitine and sphingosine may serve as potential biomarkers for CRC cells and patients with CRC. Additionally, their panel has excellent diagnostic ability for CRC. However, it must be noted that the diagnostic ability of the biomarkers we explored for CRC needs to be validated in studies with stricter designs and larger sample sizes.

## Conclusions

To the best of our knowledge, the present study was the first to validate the results of serum samples of patients with CRC using UHPLC-HRMS-based multiomics of CRC cells. The metabololite and lipid profiles of CRC cells in four different stages were successfully measured. GPL, FA, and AA were identified as the predominant differential metabolites, suggesting that their abnormal metabolism may contribute to the malignant progression of CRC. Specifically, we uncovered the key role of abnormal lipid metabolism represented by PC in CRC. The change trends of eight differential metabolites shared among the four stages of CRC cells were similar to those observed in patients with CRC. In addition, palmitoylcarnitine and sphingosine were found to be novel biomarkers for CRC cells and patients with CRC. Generally, this study offers new insight into the involvement of lipid metabolism, particularly PC metabolism, in the development of CRC. Our findings also propose a new strategy for exploring biomarkers for early detection of cancer. This in vitro strategy is a powerful supplement to the in vivo study of CRC and can improve the prevention and diagnosis of CRC. For certain, further validation in large-sized studies is needed to apply these findings in clinical settings.

### Electronic supplementary material

Below is the link to the electronic supplementary material.

Supplementary Material 1

## Data Availability

The datasets supporting the conclusions of this article are included within the article.

## Abbreviations

UHPLC-HRMS: Ultrahigh-performance liquid chromatography tandem high-resolution mass spectrometry
ESI: Electrospray ionization
ROC: The receiver operating characteristic curve
AUC: Area under the curve
NR: Normal control
CRC: Colorectal cancer
AA: Amino acid
FA: Fatty acid
GPL: Glycerophospholipid
NL: Nucleotide
OA: Organic acid
PAM: Polyamine
SM: Sphingomyelin
ST: Steroid
SPH: Sphingosine
CER: Ceramide
SUG: Sugar
LPC: Lysophosphatidylcholine
LPE: Lysophosphatidylethanolamine
LPG: Lysophosphatidylglycerol
PC: Phosphatidylcholine
PE: Phosphatidylethanolamine
