# Supplementary material for: UHPLC-HRMS-based Multiomics to Explore the Potential Mechanisms and Biomarkers for Colorectal Cancer
Source: BMC Cancer. 2024 May 27;24:644. doi: 10.1186/s12885-024-12321-7 (PMC11129395; doi:10.1186/s12885-024-12321-7)
Supplement: Supplementary file 1 — Supplementary Material 1 [file 12885_2024_12321_MOESM1_ESM.pdf]

# UHPLC-HRMS-based Multiomics to Explore the Potential Mechanisms and Biomarkers for Colorectal Cancer

Xuancheng Wang [1], Xuan Guan [1], Ying Tong [1], Yunxiao Liang [3], Zongsheng Huang [3], Mingsen Wen [1], Jichu Luo [1], Hongwei Chen [1], Shanyi Yang [1], Zhiyong She [1], Zhijuan Wei [1], Yun Zhou [1], Yali Qi [1], Pingchuan Zhu [5], Yanying Nong [2,*] and Qisong Zhang [1,4,*]

[1] Guangxi Key Laboratory of Special Biomedicine; School of Medicine, Guangxi University, Nanning, Guangxi, 530004, PR China.

[2] Department of Academic Affairs, The First Affiliated Hospital of Guangxi Medical University, Nanning, Guangxi, 530021, PR China.

[3] Department of Gastroenterology, People's Hospital of Guangxi Zhuang Autonomous Region, Nanning, Guangxi, 530021, PR China.

[4] Center for Instrumental Analysis, Guangxi University, Nanning, Guangxi, 530004, PR China.

[5] State Key Laboratory for Conservation and Utilization of Subtropical Agro-Bioresources, Guangxi University, Nanning, Guangxi, 530004, PR China.

## Supplementary Material

**Supplementary Figure S1.** The identification of differential metabolites and lipids between CRC cell groups and the control cell group.

**Supplementary Figure S2.** Clustering heatmap analysis for the level distribution of differential metabolites between the control cell group and stage A group of CRC cells.

**Supplementary Figure S3.** Clustering heatmap analysis for the level distribution of differential metabolites between the control cell group and stage B group of CRC cells.

**Supplementary Figure S4.** Clustering heatmap analysis for the level distribution of differential metabolites between the control cell group and stage C group of CRC cells.

**Supplementary Figure S5.** Clustering heatmap analysis for the level distribution of differential metabolites between the control cell group and stage D group of CRC cells.

**Supplementary Figure S6.** Clustering heatmap analysis for the level distribution of differential lipids between the control cell group and stage A group of CRC cells.

**Supplementary Figure S7.** Clustering heatmap analysis for the level distribution of differential lipids between the control cell group and stage B group of CRC cells.

**Supplementary Figure S8.** Clustering heatmap analysis for the level distribution of differential lipids between the control cell group and stage C group of CRC cells.

**Supplementary Figure S9.** Clustering heatmap analysis for the level distribution of differential lipids between the control cell group and stage D group of CRC cells.

**Supplementary Table S1.** Chromatographic conditions for cell metabolomics analysis.

**Supplementary Table S2.** Chromatographic conditions for cell lipidomics analysis.

**Supplementary Table S3.** Mass spectrometry conditions for omics analysis.

**Supplementary Table S4.** Baseline clinical characteristics of study subjects.

**Supplementary Table S5.** Compounds identification in cell metabolomics in both ESI modes.

**Supplementary Table S6.** Compounds identification in cell lipidomics in both ESI modes.

**Supplementary Table S7.** ROC analysis of common differential metabolites in four different stages of CRC cells.

**Supplementary Table S8.** ROC analysis of common differential lipids in four different stages of CRC cells.

**Supplementary Figure S1**

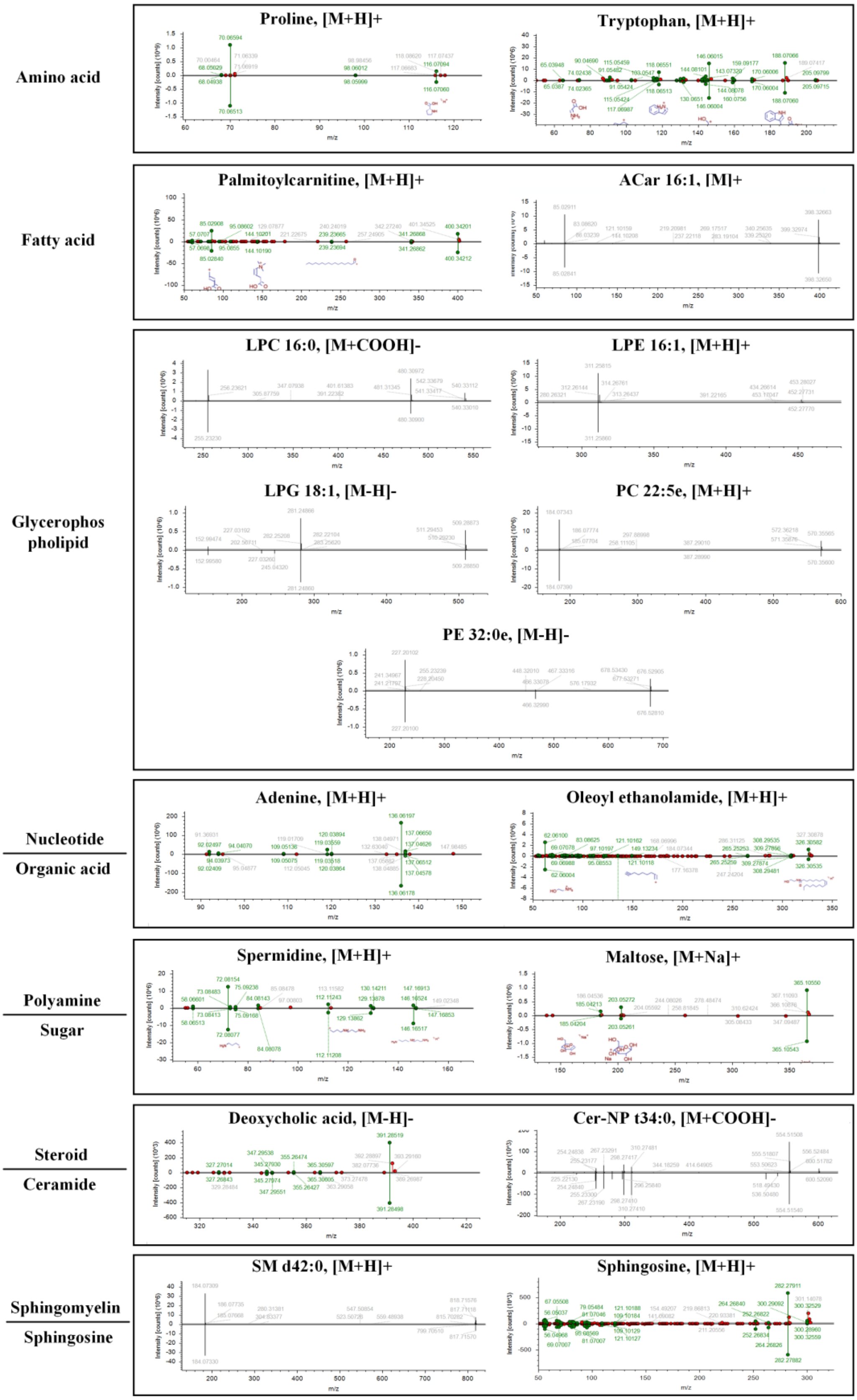


**Supplementary Figure S1.** The identification of differential metabolites and lipids between CRC cell groups and the control cell group.

**Supplementary Figure S2**

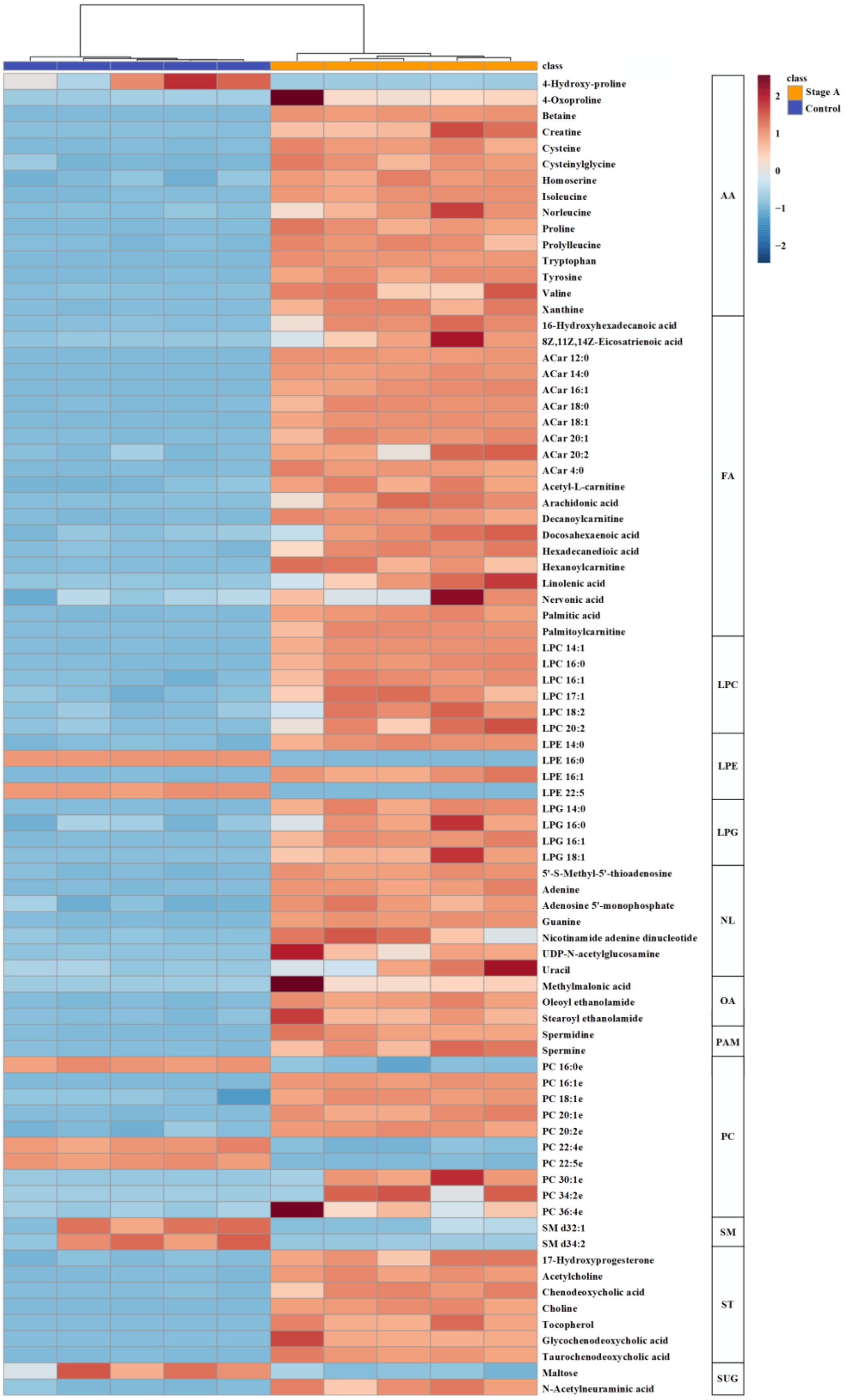


**Supplementary Figure S2.** Clustering heatmap analysis for the level distribution of differential metabolites between the control cell group and stage A group of CRC cells. Abbreviations: AA, amino acid; FA, fatty acid; LPC, lysophosphatidylcholine; LPE, lysophosphatidylethanolamine; LPG, lysophosphatidylglycerol; NL, nucleotide; OA, organic acid; PAM, polyamine; PC, phosphatidylcholine; SM, sphingomyelin; ST, steroid; SUG, sugar; ACar, acetylcarnitine.

**Supplementary Figure S3**

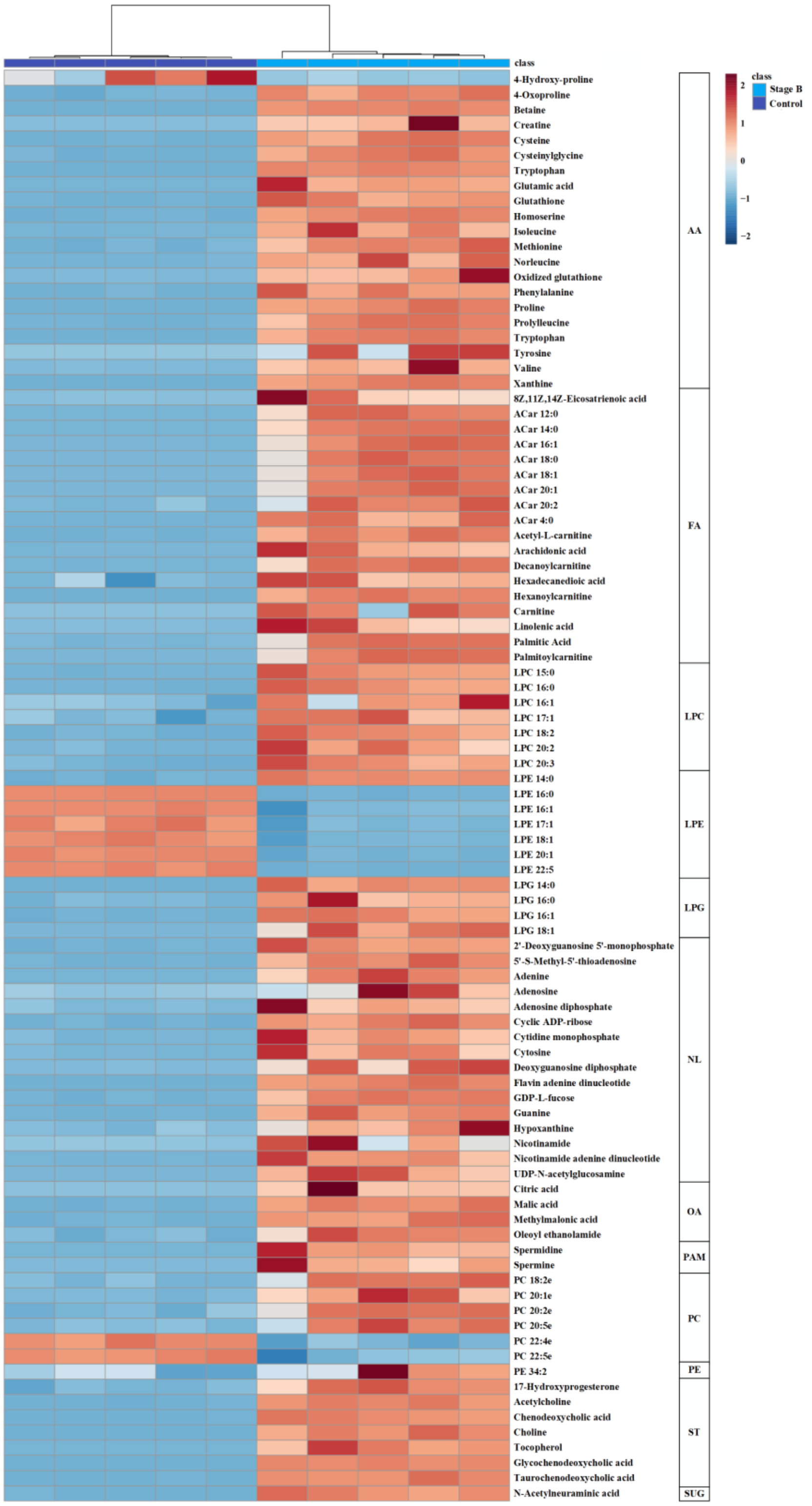


**Supplementary Figure S3.** Clustering heatmap analysis for the level distribution of differential metabolites between the control cell group and stage B group of CRC cells. Abbreviations: AA, amino acid; FA, fatty acid; LPC, lysophosphatidylcholine; LPE, lysophosphatidylethanolamine; LPG, lysophosphatidylglycerol; NL, nucleotide; OA, organic acid; PAM, polyamine; PC, phosphatidylcholine; PE, phosphatidylethanolamine; ST, steroid; SUG, sugar; ACar, acetylcarnitine.

**Supplementary Figure S4**

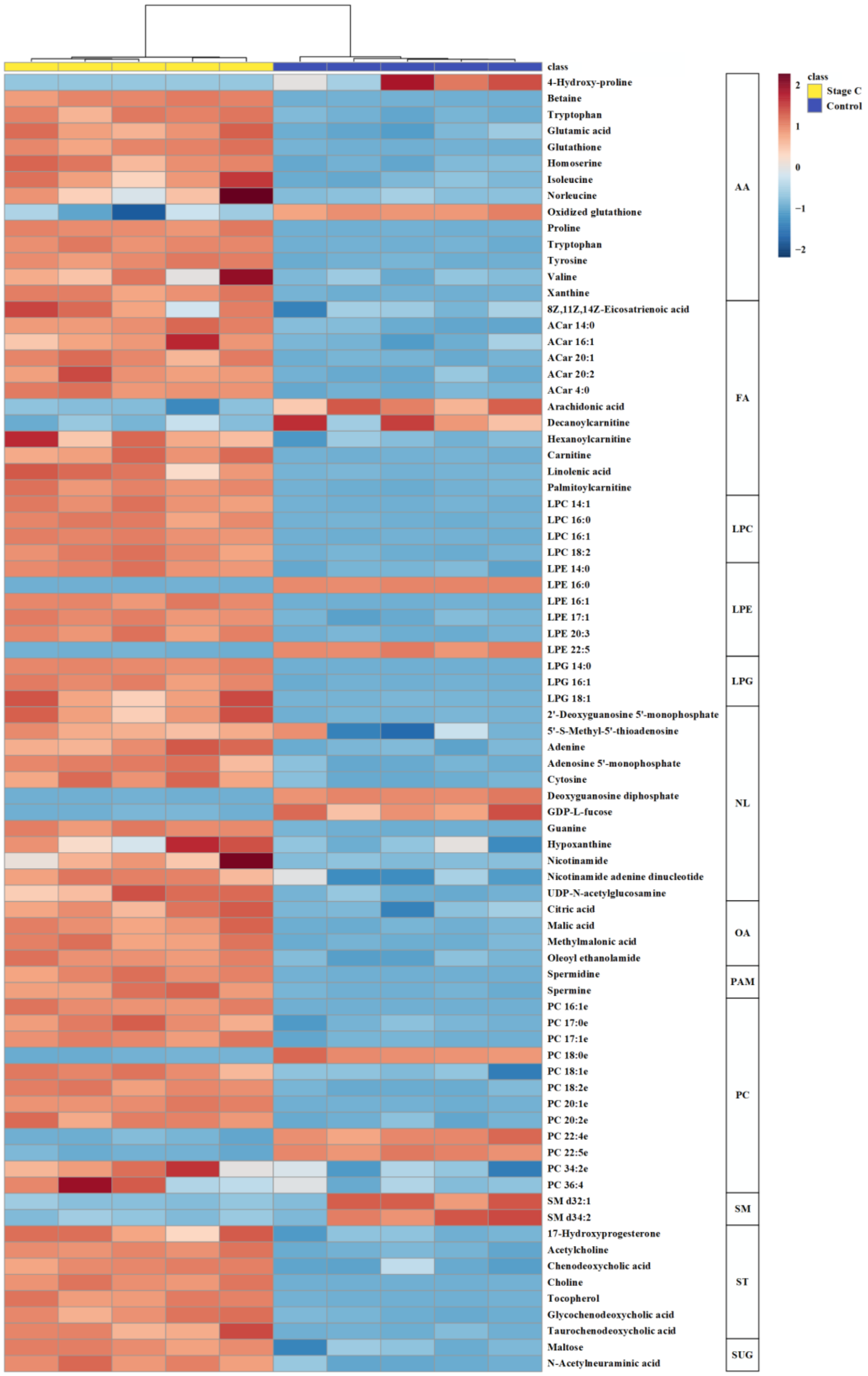


**Supplementary Figure S4.** Clustering heatmap analysis for the level distribution of differential metabolites between the control cell group and stage C group of CRC cells. Abbreviations: AA, amino acid; FA, fatty acid; LPC, lysophosphatidylcholine; LPE, lysophosphatidylethanolamine; LPG, lysophosphatidylglycerol; NL, nucleotide; OA, organic acid; PAM, polyamine; PC, phosphatidylcholine; SM, sphingomyelin; ST, steroid; SUG, sugar; ACar, acetylcarnitine.

**Supplementary Figure S5**

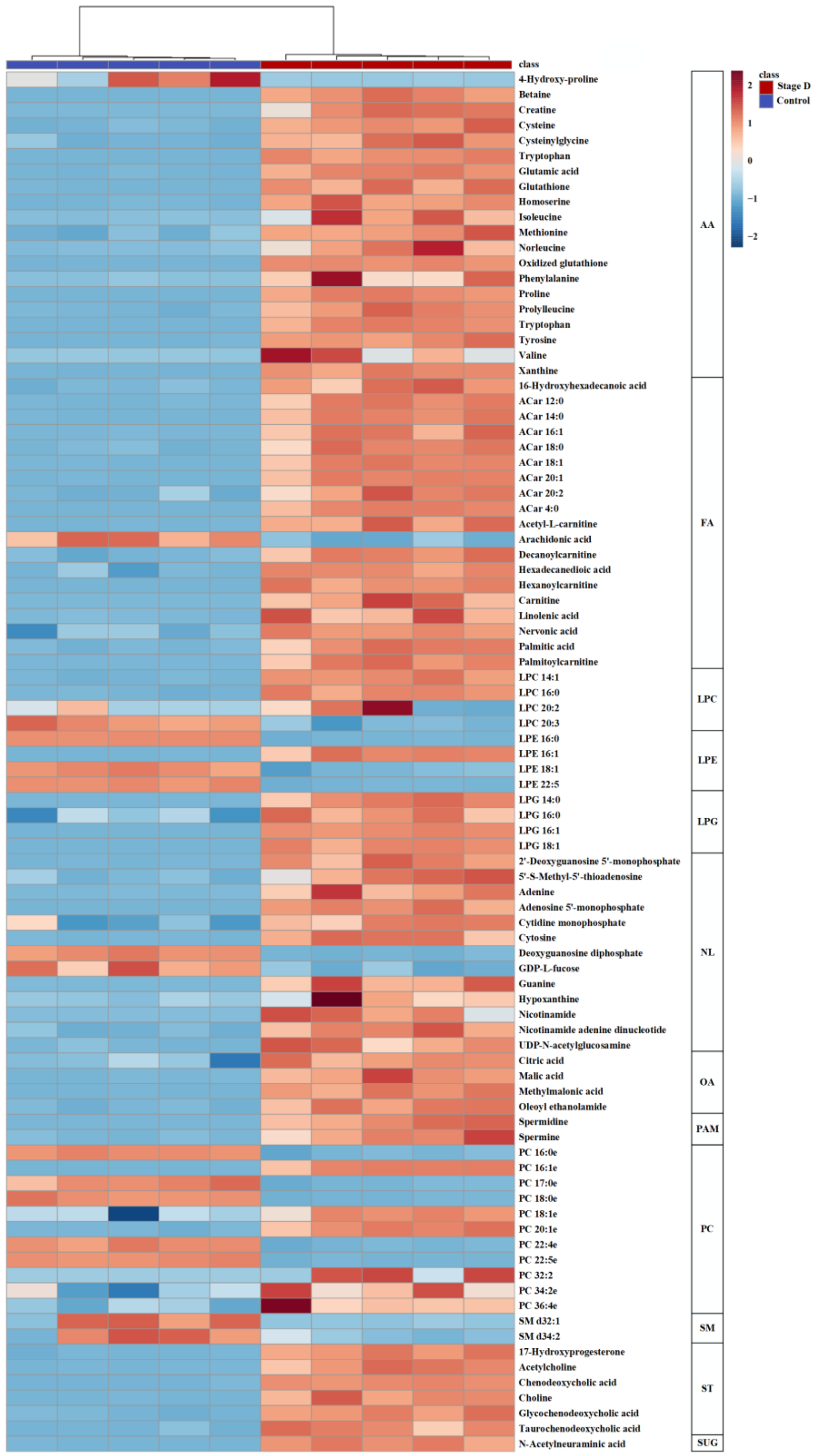


**Supplementary Figure S5.** Clustering heatmap analysis for the level distribution of differential metabolites between the control cell group and stage D group of CRC cells. Abbreviations: AA, amino acid; FA, fatty acid; LPC, lysophosphatidylcholine; LPE, lysophosphatidylethanolamine; LPG, lysophosphatidylglycerol; NL, nucleotide; OA, organic acid; PAM, polyamine; PC, phosphatidylcholine; SM, sphingomyelin; ST, steroid; SUG, sugar; ACar, acetylcarnitine.

**Supplementary Figure S6**

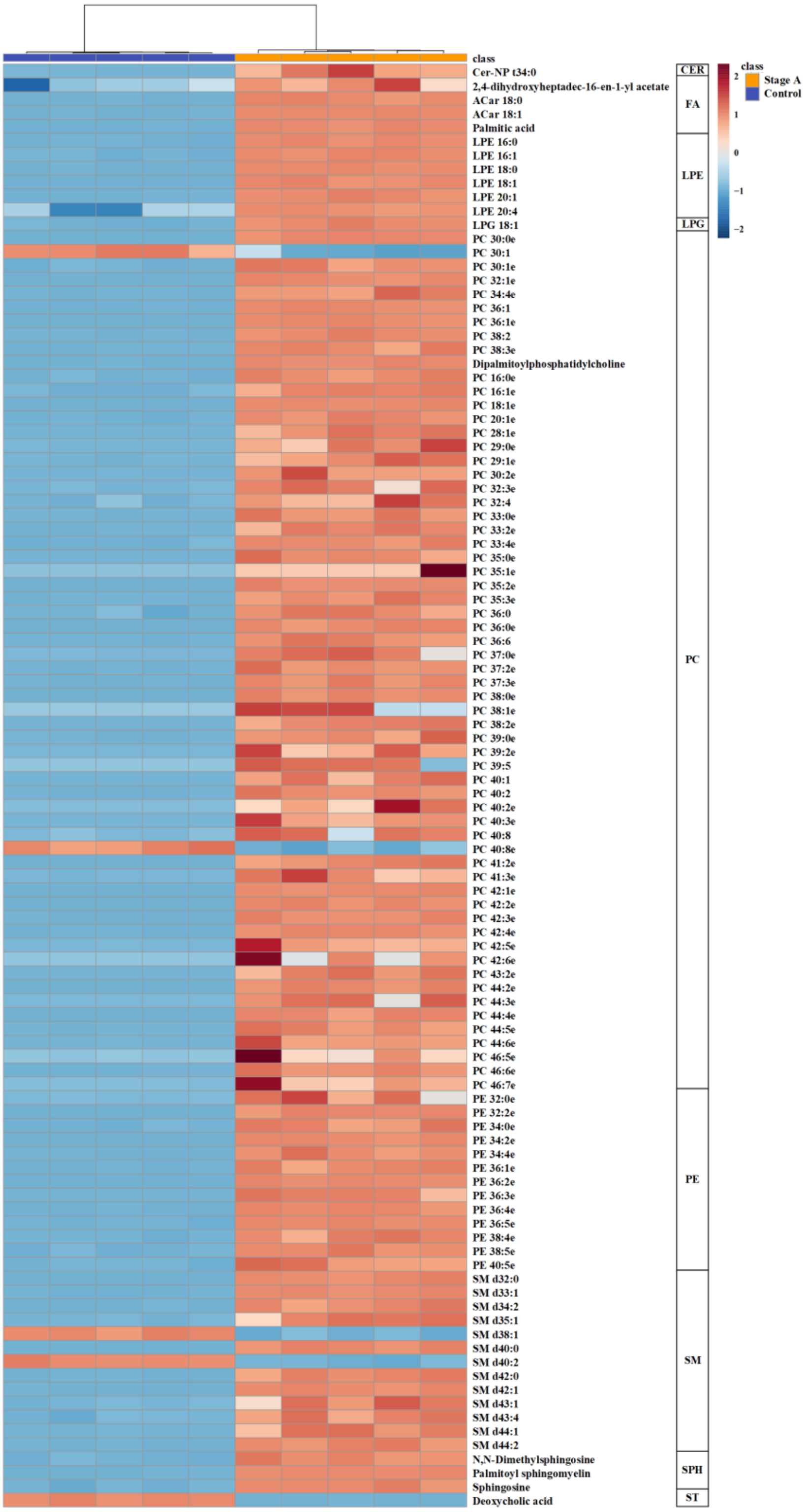


**Supplementary Figure S6.** Clustering heatmap analysis for the level distribution of differential lipids between the control cell group and stage A group of CRC cells. Abbreviations: CER, ceramide; FA, fatty acid; LPE, lysophosphatidylethanolamine; LPG, lysophosphatidylglycerol; PC, phosphatidylcholine; PE, phosphatidylethanolamine; SM, sphingomyelin; SPH, sphingosine; ST, steroid; ACar, acetylcarnitine.

**Supplementary Figure S7**

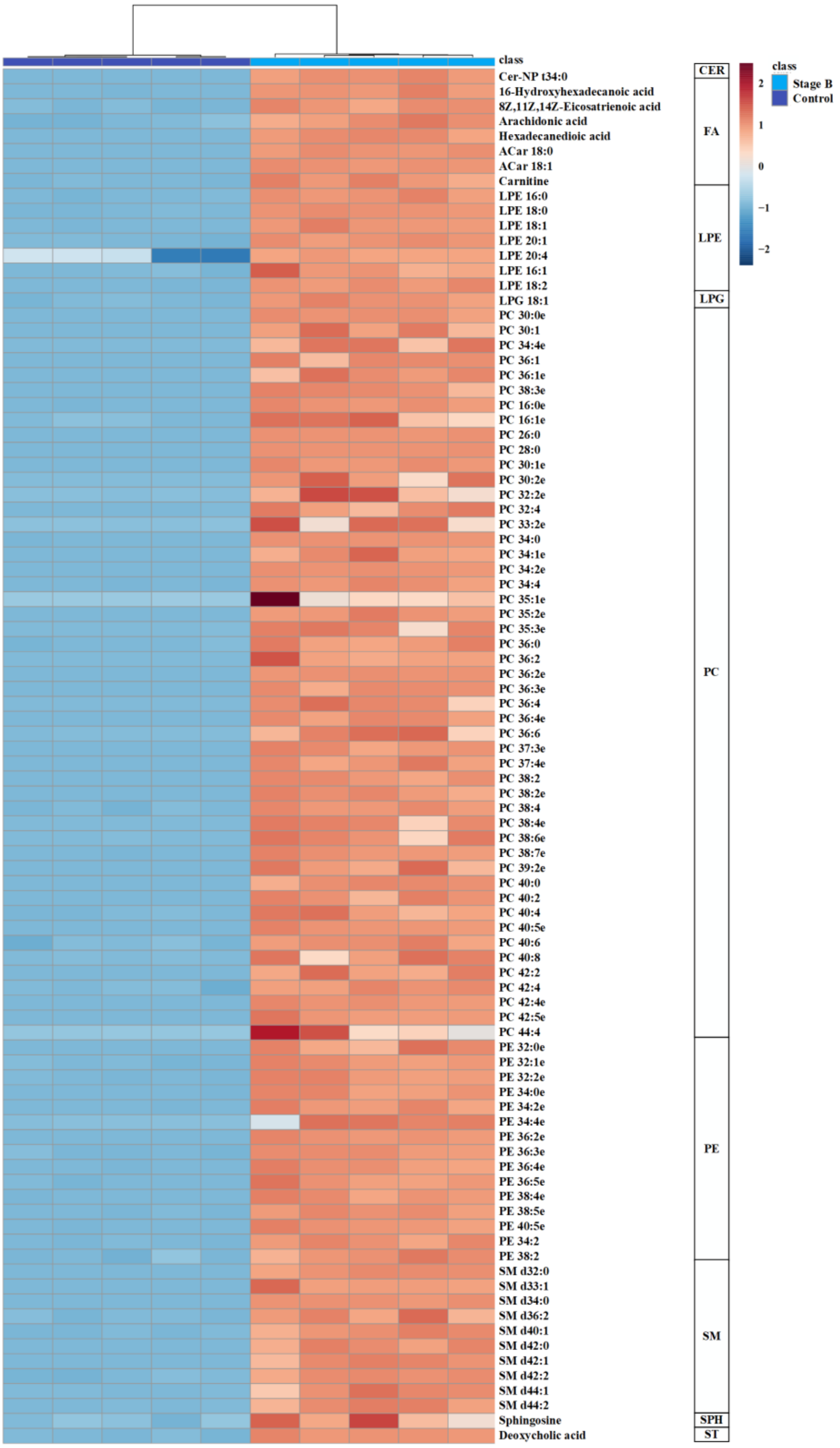


**Supplementary Figure S7.** Clustering heatmap analysis for the level distribution of differential lipids between the control cell group and stage B group of CRC cells. Abbreviations: CER, ceramide; FA, fatty acid; LPE, lysophosphatidylethanolamine; LPG, lysophosphatidylglycerol; PC, phosphatidylcholine; PE, phosphatidylethanolamine; SM, sphingomyelin; SPH, sphingosine; ST, steroid; ACar, acetylcarnitine.

**Supplementary Figure S8**

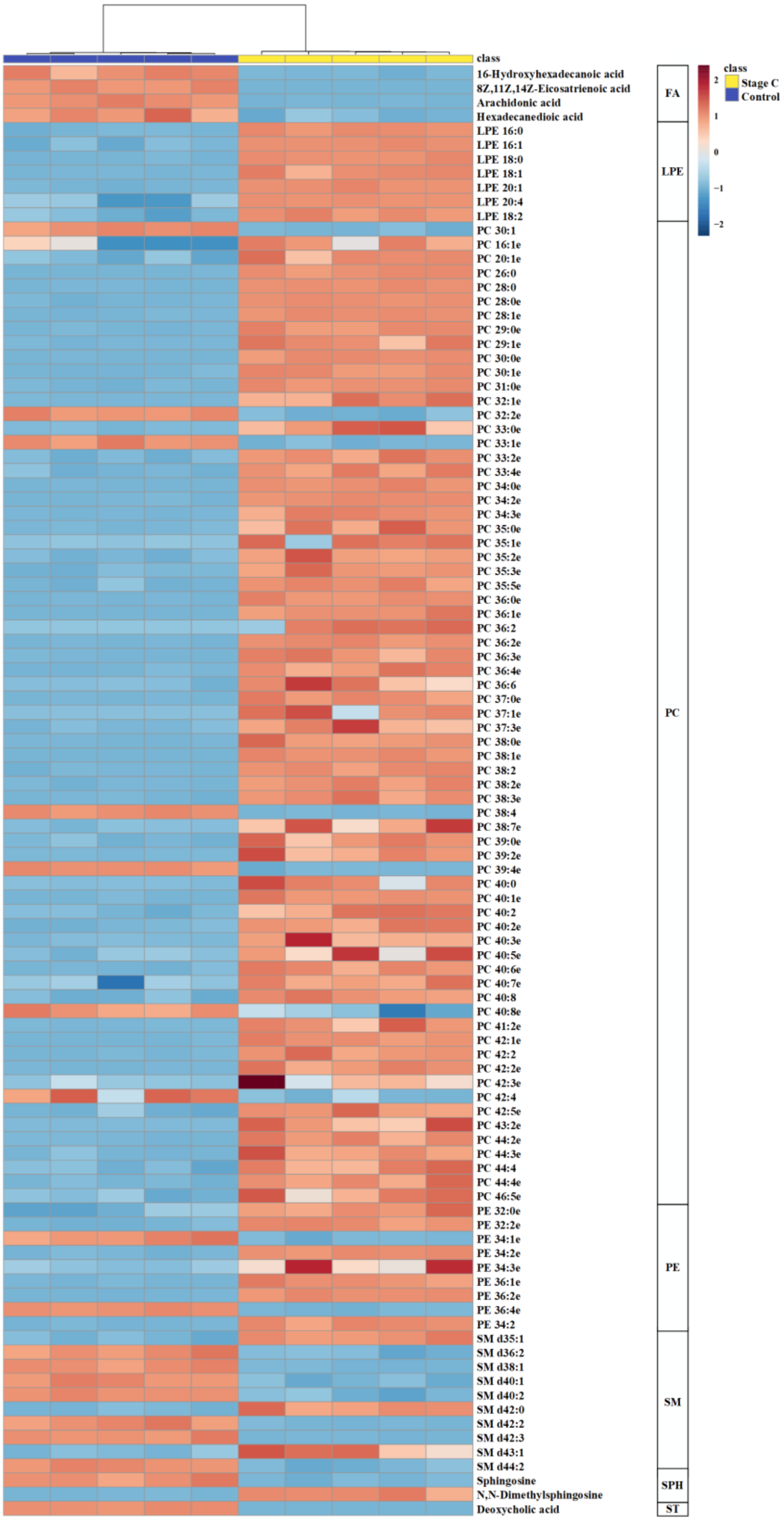


**Supplementary Figure S8.**Clustering heatmap analysis for the level distribution of differential lipids between the control cell group and stage C group of CRC cells. Abbreviations: FA, fatty acid; LPE, lysophosphatidylethanolamine; PC, phosphatidylcholine; PE, phosphatidylethanolamine; SM, sphingomyelin; SPH, sphingosine; ST, steroid.

**Supplementary Figure S9**

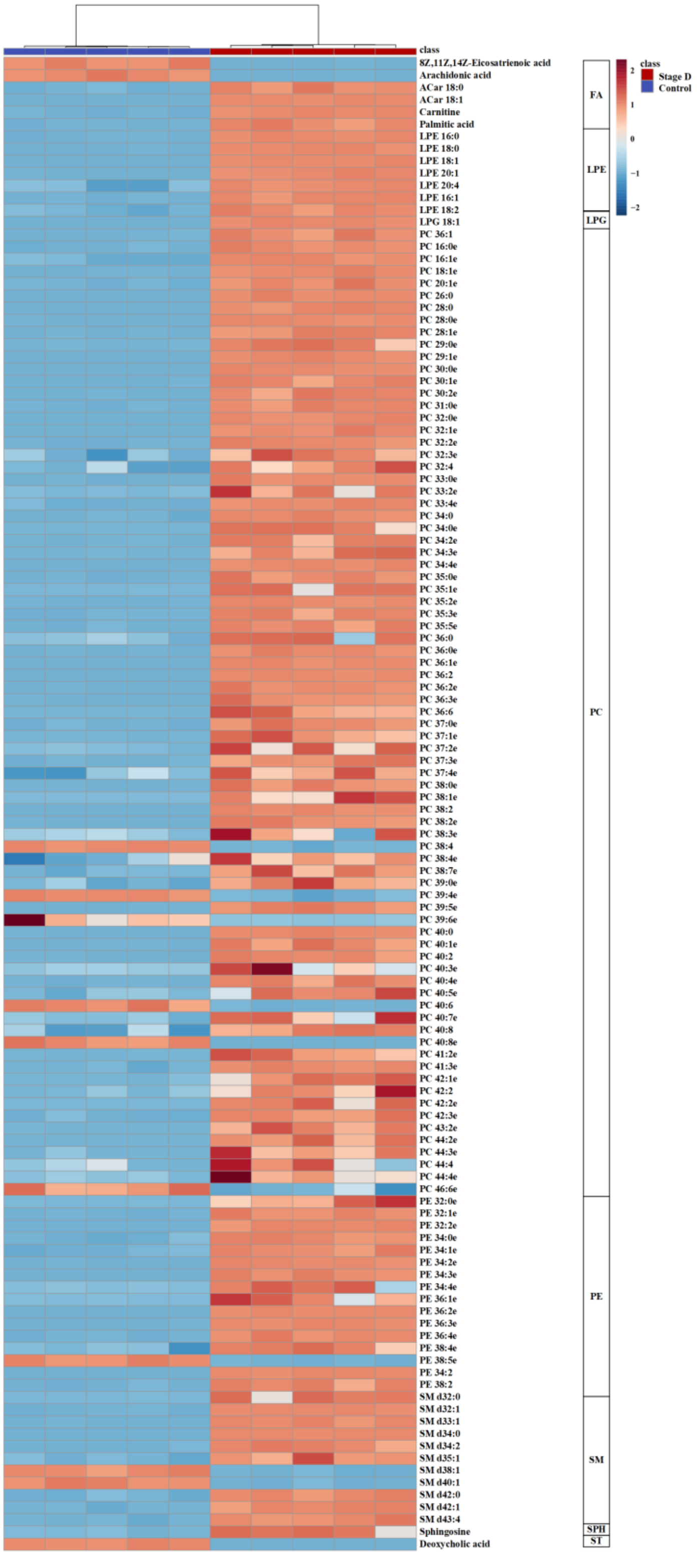


**Supplementary Figure S9.** Clustering heatmap analysis for the level distribution of differential lipids between the control cell group and stage D group of CRC cells. Abbreviations: FA, fatty acid; LPE, lysophosphatidylethanolamine; LPG, lysophosphatidylglycerol; PC, phosphatidylcholine; PE, phosphatidylethanolamine; SM, sphingomyelin; SPH, sphingosine; ST, steroid; ACar, acetylcarnitine.

**Supplementary Table S1.** Chromatographic conditions for cell metabolomics analysis.

| **Time (min)** | **A (%)** | **B (%)** | **Flow rate (mL/min)** |
|---|---|---|---|
| 0 | 98 | 2 | 0.4 |
| 4 | 98 | 2 | 0.4 |
| 15 | 0 | 100 | 0.4 |
| 20 | 0 | 100 | 0.4 |
| 22 | 98 | 2 | 0.4 |

Injection volume: 5 μL. Column temperature: 45 °C. Mobile phases: A was ultrapure water and B was acetonitrile, both of which contained formic acid (0.1% in ESI+ and 0.01% in ESI-, v/v). Notes: To avoid hindering the deprotonation of metabolites in ESI- mode, the amount of formic acid added to the mobile phase was reduced.

**Supplementary Table S2.** Chromatographic conditions for cell lipidomics analysis.

| **Time (min)** | **A (%)** | **B (%)** | **Flow rate (mL/min)** |
|---|---|---|---|
| 0 | 70 | 30 | 0.3 |
| 6 | 40 | 60 | 0.3 |
| 11 | 0 | 100 | 0.3 |
| 17 | 0 | 100 | 0.3 |
| 20 | 70 | 30 | 0.3 |

Injection volume: 5 μL. Column temperature: 50 °C. Mobile phases: A was acetonitrile and ultrapure water (6:4, v/v) and B was isopropanol and acetonitrile (9:1, v/v), both of which contained 0.1% formic acid (v/v) and 10 mM ammonium formate.

**Supplementary Table S3.** Mass spectrometry conditions for omics analysis.

| **Parameters** | **Values** |
|---|---|
| Spray voltage (kV) | 3.50 (ESI+); 2.80 (ESI-) |
| Sheath gas flow rate (psi) | 50 |

| | |
|---|---|
| Auxiliary gas flow rate (arb) | 13 |
| Capillary temperature (℃) | 320 |
| Auxiliary gas heater temperature (℃) | 420 |
| Scan range (*m/z*) | 100-1200 |
| Scan mode | Full scan (resolution of 70,000) and data-dependent MS/MS (resolution of 17,500) |

**Supplementary Table S4.** Baseline clinical characteristics of study subjects.

| | **Metabolomics** | | **Lipidomics** | |
|---|---|---|---|---|
| **Group** | NR (n=79) | CRC (n=82) | NR (n=50) | CRC (n=50) |
| **Gender (Female/Male)** | 36/43 | 43/39 | 21/29 | 23/27 |
| $P^*$ | $P$=0.38 | | $P$=0.69 | |
| **Age (year)** | 54.75 ± 12.89 | 60.36 ± 11.32 | 53.25 ± 8.01 | 61.31 ± 8.99 |
| $P^\#$ | $P$=0.55 | | $P$=0.23 | |
| **Position** | | | | |
| Rectum | - | 30 | - | 11 |
| Colon | - | 52 | - | 39 |
| **Differentiation** | | | | |
| High | - | 4 | - | 0 |
| Middle | - | 47 | - | 37 |
| Low | - | 21 | - | 6 |
| Unknown | - | 10 | - | 7 |
| **Tumor stage** | | | | |
| 0 stage | - | 3 | - | 1 |
| I stage | - | 18 | - | 11 |
| II stage | - | 35 | - | 21 |
| III stage | - | 21 | - | 15 |
| Unknown | - | 5 | - | 2 |

Abbreviations: NR, normal control; CRC, colorectal cancer. *Chi-square test and #Student's *t*-test.

**Supplementary Table S5.** Compounds identification in cell metabolomics in both ESI modes.

| ESI | No. | Name | Formula | RT (min) | Adduct | ppm | Fragments | Score |
|---|---|---|---|---|---|---|---|---|
| Neg | 1 | Linolenic acid | C18 H30 O2 | 14.44 | [M-H]- | 0.06 | 277.21732, 275.20166, 71.01236 | 92.8 |
| | 2 | 11,14-Eicosadienoic acid | C20 H36 O2 | 16.10 | [M-H]- | 0.57 | 307.26443 | 93.7 |
| | 3 | 11-Eicosenoic acid | C20 H38 O2 | 16.89 | [M-H]- | 0.99 | 309.28021 | 88.2 |
| | 4 | 11-Deoxy prostaglandin F2β | C20 H34 O4 | 13.14 | [M-H]- | 1.27 | 337.23886 | 91.8 |
| | 5 | 12-Hydroxydodecanoic acid | C12 H24 O3 | 9.53 | [M]- | 2.74 | 216.16786, 215.16473 | 84.4 |
| | 6 | 13,16-Docosadienoic Acid | C22 H40 O2 | 17.10 | [M-H]- | 1.12 | 335.29593, 96.95876, 61.98705 | 90.6 |
| | 7 | 15-OxoEDE | C20 H34 O3 | 14.36 | [M-H]- | 0.88 | 321.24380 | 89.7 |
| | 8 | 16-Hydroxyhexadecanoic acid | C16 H32 O3 | 12.67 | [M-H]- | 0.82 | 271.22809, 253.21693, 225.22240 | 94.9 |
| | 9 | 20-Hydroxyeicosatetraenoic acid | C20 H32 O3 | 14.03 | [M-H]- | 0.37 | 319.22775, 217.19627 | 86.3 |
| | 10 | 2'-Deoxyguanosine 5'-monophosphate | C10 H14 N5 O7 P | 0.71 | [M-H]- | 0.73 | 346.05606, 211.00111, 96.96820 | 74.3 |
| | 11 | 3-Hydroxyanthranilic acid | C7 H7 N O3 | 8.74 | [M-H]- | 6.36 | 152.03435 | 81.7 |
| | 12 | 4-Oxoproline | C5 H7 N O3 | 0.75 | [M-H]- | 9.35 | 128.03412, 82.02862 | 96.1 |
| | 13 | 8Z,11Z,14Z-Eicosatrienoic acid | C20 H34 O2 | 15.45 | [M-H]- | 0.58 | 305.24878 | 95.9 |
| | 14 | Arachidonic acid | C20 H32 O2 | 14.98 | [M-H]- | 0.35 | 303.23306, 259.24243, 205.19560 | 93.3 |
| | 15 | Chenodeoxycholic acid | C24 H40 O4 | 11.57 | [M-H]- | 0.76 | 391.28568, 373.27591 | 90.7 |

| | | | | | | | |
|---|---|---|---|---|---|---|---|
| 16 | Citric acid | C6 H8 O7 | 0.74 | [M-H]- | 5.42 | 191.01869, 111.00743, 87.00748 | 96.8 |
| 17 | Cyclic ADP-ribose | C15 H21 N5 O13 P2 | 0.71 | [M-H]- | 0.46 | 540.05408, 328.04553, 158.92424 | 74.1 |
| 18 | Phenyllactic acid | C9 H10 O3 | 6.40 | [M-H]- | 6.17 | 147.04413, 119.04893, 72.99180 | 90.6 |
| 19 | Tryptophan | C11 H12 N2 O2 | 4.52 | [M-H]- | 1.78 | 203.08224, 142.06535, 74.02340 | 95.8 |
| 20 | Deoxycholic Acid | C24 H40 O4 | 11.77 | [M-H]- | 0.76 | 391.28568, 365.30774, 327.26846 | 91.6 |
| 21 | Deoxyguanosine diphosphate | C10 H15 N5 O10 P2 | 0.76 | [M-H]- | 0.9 | 426.02252, 290.90808, 134.04596 | 79.4 |
| 22 | Malic acid | C4 H6 O5 | 0.73 | [M-H]- | 9.0 | 133.01305, 115.00243, 71.01247 | 90.9 |
| 23 | Leucine | C6 H13 N O2 | 1.13 | [M-H]- | 9.09 | 130.08617 | 98.6 |
| 24 | Docosahexaenoic acid | C22 H32 O2 | 14.80 | [M-H]- | 1.06 | 327.23331, 283.24338, 121.10036 | 89.2 |
| 25 | Flavin adenine dinucleotide (FAD) | C27 H33 N9 O15 P2 | 5.26 | [M-H]- | 1.69 | 784.15118, 437.08637, 346.05603 | 93.0 |
| 26 | Glycochenodeoxycholic acid | C26 H43 N O5 | 10.25 | [M-H]- | 0.90 | 448.30725, 74.02336 | 87.8 |
| 27 | Hexadecanedioic acid | C16 H30 O4 | 11.76 | [M-H]- | 1.25 | 285.20749, 267.19681, 223.20622 | 93.4 |
| 28 | Lauric acid | C12 H24 O2 | 13.28 | [M-H]- | 2.78 | 199.16980, 59.01275 | 79.7 |
| 29 | L-Glutathione oxidized | C20 H32 N6 O12 S2 | 0.74 | [M-H]- | 0.84 | 611.14417, 306,07678, 272.08838 | 85.3 |
| 30 | Linoleic acid | C18 H32 O2 | 15.21 | [M-H]- | 0.49 | 279.23309 | 95.1 |
| 31 | LPC 14:0 | C22 H46 N O7 P | 11.12 | [M+COOH]- | 0.44 | 512.29962, 452.27872, 277.20119 | 82.6 |
| 32 | LPC 14:1 | C22 H44 N O7 P | 10.20 | [M+COOH]- | 0.68 | 510.29013, 450.26321, 225.18559 | 77.7 |

| | | | | | | | |
|---|---|---|---|---|---|---|---|
| 33 | LPC 15:0 | C23 H48 N O7 P | 11.69 | [M+COOH]- | 8.51 | 526.31952, 466.29407, 241.21701 | 82.8 |
| 34 | LPC 16:0 | C24 H50 N O7 P | 12.29 | [M+COOH]- | 0.79 | 540.33112, 480.30972 | 76.0 |
| 35 | LPC 16:1 | C24 H48 N O7 P | 11.27 | [M+COOH]- | 0.94 | 538.31555, 478.29428, 253.21718 | 75.5 |
| 36 | LPC 17:1 | C25 H50 N O7 P | 12.05 | [M+COOH]- | 0.00 | 552.33069, 492.30991, 267.23297 | 77.8 |
| 37 | LPC 18:2 | C26 H50 N O7 P | 11.69 | [M+COOH]- | 0.97 | 564.33124, 504.31009, 279.23303 | 81.1 |
| 38 | LPC 18:3 | C26 H48 N O7 P | 11.29 | [M+COOH]- | 0.37 | 562.31525, 502.29431, 277.21660 | 72.7 |
| 39 | LPC 20:2 | C28 H54 N O7 P | 13.08 | [M+COOH]- | 0.64 | 592.36237, 532.34100, 307.26431 | 81.1 |
| 40 | LPC 20:3 | C28 H52 N O7 P | 12.33 | [M+COOH]- | 5.43 | 590.34955, 530.32544, 305.24869 | 74.0 |
| 41 | LPE 14:0 | C19 H40 N O7 P | 11.06 | [M-H]- | 0.89 | 424.24734, 227.20119, 196.03717 | 74.2 |
| 42 | LPE 20:4 | C25 H44 N O7 P | 11.88 | [M-H]- | 1.03 | 500.27878, 303.23303, 214.04803 | 75.7 |
| 43 | LPE 22:3 | C27 H50 N O7 P | 13.49 | [M-H]- | 0.72 | 530.32483, 333.28009, 196.03741 | 72.7 |
| 44 | LPE 22:4 | C27 H48 N O7 P | 12.58 | [M-H]- | 0.83 | 528.31000, 331.26443 | 74.6 |
| 45 | LPG 14:0 | C20 H41 O9 P | 12.57 | [M-H]- | 1.29 | 455.24213, 227.20111, 152.99500 | 70.7 |
| 46 | LPG 16:0 | C22 H45 O9 P | 15.87 | [M-H]- | 0.48 | 483.27261, 255.23283, 152.99496 | 72.5 |
| 47 | LPG 16:1 | C22 H43 O9 P | 13.25 | [M-H]- | 0.66 | 481.25751, 253.21721, 152.99472 | 71.0 |
| 48 | LPG 18:1 | C24 H47 O9 P | 15.89 | [M-H]- | 0.47 | 509.28873, 281.24866, 152.99474 | 72.0 |
| 49 | Phenylalanine | C9 H11 N O2 | 0.76 | [M-H]- | 6.72 | 164.07059, 147.04382, 72.00777 | 94.8 |

| | | | | | | | | |
|---|---|---|---|---|---|---|---|---|
| | 50 | Methylmalonic acid | C4 H6 O4 | 0.75 | [M-H]- | 9.25 | 117.01825, 73.02811 | 94.1 |
| | 51 | Myristic acid | C14 H28 O2 | 14.56 | [M-H]- | 1.91 | 227.20122 | 96.4 |
| | 52 | Nervonic acid | C24 H46 O2 | 19.63 | [M-H]- | 0.56 | 365.34271 | 99.1 |
| | 53 | Oleic acid | C18 H34 O2 | 15.89 | [M-H]- | 0.41 | 281.24872 | 93.4 |
| | 54 | Palmitoleic acid | C16 H30 O2 | 14.82 | [M-H]- | 0.18 | 253.21735 | 96.3 |
| | 55 | PE 34:2 | C39 H74 N O8 P | 15.89 | [M-H]- | 0.95 | 714.50861, 281.24863, 253.21730 | 70.5 |
| | 56 | Pentadecanoic acid | C15 H30 O2 | 14.98 | [M-H]- | 0.11 | 241.21733 | 86.6 |
| | 57 | Perillic acid | C10 H14 O2 | 9.26 | [M-H]- | 5.83 | 165.09114 | 82.6 |
| | 58 | SM d32:1 | C37 H75 N2 O6 P | 14.88 | [M+COOH]- | 1.18 | 719.53363, 659.51422, 168.04222 | 77.3 |
| | 59 | Stearic acid | C18 H36 O2 | 17.11 | [M-H]- | 0.65 | 283.26407 | 95.7 |
| | 60 | Taurochenodeoxycholic acid | C26 H45 N O6 S | 9.87 | [M-H]- | 1.36 | 498.29016 | 97.9 |
| | 61 | Tetradecanedioic acid | C14 H26 O4 | 10.54 | [M-H]- | 0.09 | 257.17581, 239.16512, 195.17456 | 82.5 |
| | 62 | UDP-N-acetylglucosamine | C17 H27 N3 O17 P2 | 0.69 | [M-H]- | 0.89 | 606.07483, 508.10291, 362.00485 | 93.6 |
| | 63 | Xanthine | C5 H4 N4 O2 | 0.75 | [M-H]- | 6.55 | 151.02516, 108.01907 | 78.1 |
| **Pos** | 64 | 17-Hydroxyprogesterone | C21 H30 O3 | 13.77 | [M+H]+ | 8.64 | 331.22391, 119.08592, 95.08618 | 73.5 |
| | 65 | N-Valylphenylalanine | C14 H20 N2 O3 | 4.80 | [M+H]+ | 1.25 | 265.15500, 166.08627, 72.08157 | 95.7 |
| | 66 | 2-Arachidonoyl glycerol | C23 H38 O4 | 12.76 | [M+H]+ | 7.08 | 379.28156, 123.11633, 85.10169 | 85.9 |

| | | | | | | | |
|---|---|---|---|---|---|---|---|
| 67 | 5'-S-Methyl-5'-thioadenosine | C11 H15 N5 O3 S | 0.80 | [M+H]+ | 1.22 | 298.09720, 163.04218, 136.06192 | 98.5 |
| 68 | 9-Oxo-10,12-octadecadienoic acid | C18 H30 O3 | 13.08 | [M+H]+ | 1.94 | 195.22620, 277.21652, 151.11209 | 89.3 |
| 69 | ACar 12:0 | C19 H38 N O4 | 10.27 | [M]+ | 1.06 | 344.27972, 85.02912 | 77.7 |
| 70 | ACar 14:0 | C21 H42 N O4 | 11.26 | [M]+ | 0.84 | 372.31107, 85.02913 | 74.4 |
| 71 | ACar 16:1 | C23 H44 N O4 | 11.54 | [M]+ | 1.01 | 398.32663, 85.02911 | 76.7 |
| 72 | ACar 18:0 | C25 H50 N O4 | 13.05 | [M]+ | 0.69 | 428.37369, 85.02912 | 71.8 |
| 73 | ACar 18:1 | C25 H48 N O4 | 12.42 | [M]+ | 0.64 | 426.35806, 85.02912 | 87.8 |
| 74 | ACar 20:1 | C27 H52 N O4 | 13.19 | [M]+ | 2.45 | 454.38852, 85.02909 | 84.7 |
| 75 | ACar 20:2 | C27 H50 N O4 | 12.66 | [M]+ | 0.19 | 452.37390, 85.02917 | 70.5 |
| 76 | ACar 4:0 | C11 H22 N O4 | 4.49 | [M]+ | 2.51 | 232.15430, 85.02906 | 73.7 |
| 77 | Acetylcholine | C7 H15 N O2 | 0.66 | [M+H]+ | 1.13 | 146.11772, 87.04474, 60.08168 | 87.7 |
| 78 | Acetyl-L-carnitine | C9 H17 N O4 | 0.76 | [M+H]+ | 1.06 | 204.12325, 144.10201, 85.02911 | 96.6 |
| 79 | Adenine | C5 H5 N5 | 0.73 | [M+H]+ | 1.46 | 136.06197, 119.03559, 92.02497 | 95 |
| 80 | Adenosine | C10 H13 N5 O4 | 1.89 | [M+H]+ | 0.34 | 268.10394, 136.06184, 85.02914 | 98.8 |
| 81 | Adenosine 5'-monophosphate | C10 H14 N5 O7 P | 0.72 | [M+H]+ | 0.32 | 348.07025, 136.06186, 97.02888 | 99.4 |
| 82 | Adenosine diphosphate | C10 H15 N5 O10 P2 | 0.70 | [M+H]+ | 0.98 | 428.03711, 330.06094, 136.06195 | 96.1 |
| 83 | Betaine | C5 H11 N O2 | 0.63 | [M+H]+ | 2.92 | 118.08662, 60.0772, 58.06605 | 70.3 |

| | | | | | | | |
|---|---|---|---|---|---|---|---|
| 84 | Choline | C5 H13 N O | 0.61 | [M+H]+ | 3.65 | 104.10737, 60.08162, 58.06598 | 86.9 |
| 85 | Cortisone | C21 H28 O5 | 8.26 | [M+H]+ | 0.44 | 361.20111, 163.11188, 121.06519 | 87.0 |
| 86 | Creatine | C4 H9 N3 O2 | 0.66 | [M+H]+ | 1.34 | 132.07693, 114.06655, 90.05558 | 99.3 |
| 87 | Creatinine | C4 H7 N3 O | 0.38 | [M+H]+ | 3.17 | 114.06655, 86.07203, 72.04511 | 86.0 |
| 88 | Cysteinylglycine | C5 H10 N2 O3 S | 0.68 | [M]+ | 0.07 | 179.04842, 116.01681, 76.02231 | 86.0 |
| 89 | Cytidine monophosphate | C9 H14 N3 O8 P | 0.72 | [M+H]+ | 2.97 | 324.06009, 112.05101, 97.02898 | 98.0 |
| 90 | Cytosine | C4 H5 N3 O | 0.72 | [M+H]+ | 4.12 | 112.05108, 95.02467, 67.03005 | 90.2 |
| 91 | Maltose | C12 H22 O11 | 0.68 | [M+Na]+ | 0.18 | 365.10550, 203.05272, 185.04213 | 93.2 |
| 92 | Proline | C5 H9 N O2 | 0.66 | [M+H]+ | 2.89 | 116.07094, 98.06012, 70.06594 | 98.8 |
| 93 | Cysteine | C3 H7 N O2 S | 0.64 | [M+H]+ | 2.82 | 105.00101, 76.002235, 58.99593 | 81.6 |
| 94 | Decanoylcarnitine | C17 H33 N O4 | 9.21 | [M+H]+ | 0.65 | 316.24844, 257.17465, 85.02911 | 95.9 |
| 95 | Homoserine | C4 H9 N O3 | 0.61 | [M+H]+ | 2.92 | 120.06587, 102.05549, 74.06086 | 70.8 |
| 96 | Tryptophan | C11 H12 N2 O2 | 4.85 | [M+H]+ | 4.08 | 205.09799, 188.07066, 146.06015 | 93.2 |
| 97 | D-α-Tocopherol | C29 H50 O2 | 14.57 | [M+H]+ | 0.11 | 431.38831, 165.09111, 111.11729 | 93.7 |
| 98 | Guanine | C5 H5 N5 O | 0.76 | [M+H]+ | 2.52 | 152.05707, 110.03535, 80.02495 | 87.7 |
| 99 | Hexadecanamide | C16 H33 N O | 14.73 | [M+H]+ | 0.59 | 256.26334, 116.10760, 88.07632 | 98.0 |
| 100 | Hexanoylcarnitine | C13 H25 N O4 | 6.50 | [M+H]+ | 0.48 | 260.18576, 144.10205, 85.02913 | 98.1 |

| | | | | | | | |
|---|---|---|---|---|---|---|---|
| 101 | Hypoxanthine | C5 H4 N4 O | 2.18 | [M+H]+ | 0.68 | 137.04588, 94.04060, 67.03003 | 92.2 |
| 102 | Isoleucine | C6 H13 N O2 | 1.48 | [M+H]+ | 1.02 | 132.10204, 86.09705, 69.07066 | 99.0 |
| 103 | Carnitine | C7 H15 N O3 | 0.63 | [M+H]+ | 0.74 | 162.11259, 103.03947, 60.08169 | 96.6 |
| 104 | Methionine | C5 H11 N O2 S | 0.76 | [M+H]+ | 0.36 | 150.05838, 104.05335, 61.01156 | 93.5 |
| 105 | Glutamic acid | C5 H9 N O4 | 0.62 | [M+H]+ | 0.98 | 148.06058, 102.05546, 84.04510 | 97.5 |
| 106 | L-Glutathione (reduced) | C10 H17 N3 O6 S | 0.76 | [M+H]+ | 0.17 | 308.09103, 179.04863, 76.02233 | 92.8 |
| 107 | Linoleoyl ethanolamide | C20 H37 N O2 | 13.71 | [M+H]+ | 1.22 | 324.29010, 95.08611, 62.06095 | 89.6 |
| 108 | Norleucine | C6 H13 N O2 | 1.62 | [M+H]+ | 1.02 | 132.10204, 86.09705, 69.07066 | 99.6 |
| 109 | LPE 16:0 | C21 H44 N O7 P | 12.04 | [M+H]+ | 0.27 | 454.29294, 313.27380 | 71.2 |
| 110 | LPE 16:1 | C21 H42 N O7 P | 11.26 | [M+H]+ | 0.32 | 452.27731, 311.25815 | 74.4 |
| 111 | LPE 17:1 | C22 H44 N O7 P | 11.82 | [M+H]+ | 0.01 | 466.29282, 325.27383 | 70.2 |
| 112 | LPE 18:1 | C23 H46 N O7 P | 12.41 | [M+H]+ | 0.07 | 480.30850, 339.28943 | 70.5 |
| 113 | LPE 20:1 | C25 H50 N O7 P | 13.55 | [M+H]+ | 0.13 | 508.33972, 367.32083 | 72.8 |
| 114 | LPE 20:3 | C25 H46 N O7 P | 12.14 | [M+H]+ | 0.13 | 504.30853, 363.28934 | 71.4 |
| 115 | LPE 22:5 | C27 H46 N O7 P | 12.33 | [M+H]+ | 0.94 | 528.30896, 387.28955 | 70.2 |
| 116 | Tyrosine | C9 H11 N O3 | 0.77 | [M+H]+ | 1.65 | 182.08147, 136.07587, 91.05488 | 89.1 |
| 117 | N-Acetylneuraminic acid | C11 H19 N O9 | 0.66 | [M+Na]+ | 0.50 | 332.09537, 314.08484, 211.05795 | 95.3 |

| | | | | | | | |
|---|---|---|---|---|---|---|---|
| 118 | Nicotinamide adenine dinucleotide | C21 H27 N7 O14 P2 | 0.72 | [M+H]+ | 0.28 | 664.11621, 428.03656, 136.06187 | 94.8 |
| 119 | Oleamide | C18 H35 N O | 15.02 | [M+H]+ | 0.46 | 282.27927, 149.13290, 83.08627 | 96.9 |
| 120 | Oleoyl ethanolamide | C20 H39 N O2 | 14.50 | [M+H]+ | 1.42 | 326.30582, 265.25253, 62.06100 | 95.1 |
| 121 | Palmitic acid | C16 H32 O2 | 9.65 | [M+NH4]+ | 0.64 | 274.27423, 212.23581, 102.09192 | 85.4 |
| 122 | Palmitoyl sphingomyelin | C39 H79 N2 O6 P | 21.73 | [M+H]+ | 3.05 | 703.57697, 184.07353, 86.09714 | 95.1 |
| 123 | Palmitoylcarnitine | C23 H45 N O4 | 12.19 | [M+H]+ | 0.31 | 400.34201, 239.23665, 85.02908 | 95.4 |
| 124 | PC 16:0e | C24 H50 N O7 P | 12.10 | [M+H]+ | 0.15 | 496.33969, 313.27316, 184.07341 | 83.7 |
| 125 | PC 16:1e | C24 H48 N O7 P | 11.31 | [M+H]+ | 0.09 | 494.32416, 311.25806, 184.07343 | 85.2 |
| 126 | PC 17:0e | C25 H52 N O7 P | 12.50 | [M+H]+ | 0.34 | 510.35559, 327.28891, 184.07336 | 84.9 |
| 127 | PC 17:1e | C25 H50 N O7 P | 11.88 | [M+H]+ | 0.20 | 508.33987, 325.27365, 184.07339 | 87.1 |
| 128 | PC 18:0e | C26 H54 N O7 P | 13.34 | [M+H]+ | 0.29 | 524.37122, 341.30478, 184.07343 | 85.2 |
| 129 | PC 18:1e | C26 H52 N O7 P | 12.41 | [M+H]+ | 0.10 | 522.35547, 339.29034, 184.07341 | 85.7 |
| 130 | PC 18:2e | C26 H50 N O7 P | 11.73 | [M+H]+ | 0.85 | 520.34021, 337.27356, 184.07349 | 86.9 |
| 131 | PC 20:1e | C28 H56 N O7 P | 13.60 | [M+H]+ | 0.23 | 550.38684, 184.07344 | 85.6 |
| 132 | PC 20:2e | C28 H54 N O7 P | 12.80 | [M+H]+ | 0.61 | 548.37140, 184.07350 | 87.2 |
| 133 | PC 20:5e | C28 H48 N O7 P | 11.22 | [M+H]+ | 1.08 | 542.32471, 184.07355 | 84.3 |
| 134 | PC 22:4e | C30 H54 N O7 P | 12.67 | [M+H]+ | 1.13 | 572.37042, 184.07344 | 83.2 |

| | | | | | | | |
|---|---|---|---|---|---|---|---|
| 135 | PC 22:5e | C30 H52 N O7 P | 12.06 | [M+H]+ | 0.41 | 570.35565, 387.29010, 184.07343 | 86.7 |
| 136 | PC 28:0 | C36 H72 N O8 P | 15.63 | [M+H]+ | 0.05 | 678.50684, 184.07341 | 75.1 |
| 137 | PC 28:0 | C36 H74 N O7 P | 15.89 | [M+H]+ | 1.13 | 664.52832, 481.35522, 184.07352 | 88.9 |
| 138 | PC 30:0e | C38 H78 N O7 P | 15.81 | [M+H]+ | 3.84 | 692.55621, 184.07344 | 88.7 |
| 139 | PC 30:1e | C38 H76 N O7 P | 15.93 | [M+H]+ | 0.01 | 690.54321, 184.07347 | 90.7 |
| 140 | PC 32:2 | C40 H76 N O8 P | 15.18 | [M+H]+ | 0.52 | 730.53851, 184.07350 | 74.7 |
| 141 | PC 34:2 | C42 H80 N O8 P | 16.36 | [M+H]+ | 0.35 | 758.56970, 184.07346 | 75.4 |
| 142 | PC 34:2e | C42 H82 N O7 P | 21.66 | [M+H]+ | 0.38 | 744.59045, 184.07353 | 88.6 |
| 143 | PC 36:4 | C44 H80 N O8 P | 16.75 | [M+H]+ | 0.19 | 782.56958, 184.07349 | 74.1 |
| 144 | PC 36:4e | C44 H82 N O7 P | 15.88 | [M+H]+ | 3.52 | 768.58746, 184.07352 | 72.5 |
| 145 | PC 40:6 | C48 H84 N O8 P | 16.41 | [M+H]+ | 0.10 | 834.60065, 184.07350 | 73.4 |
| 146 | Phenylacetylglycine | C10 H11 N O3 | 5.91 | [M+H]+ | 3.09 | 194.08057, 91.05482, 76.04005 | 92.8 |
| 147 | Prolylleucine | C11 H20 N2 O3 | 4.61 | [M+H]+ | 4.15 | 229.15562, 70.06591 | 83.5 |
| 148 | Propionylcarnitine | C10 H19 N O4 | 2.03 | [M+H]+ | 0.85 | 218.13850, 85.02905, 60.08159 | 83.9 |
| 149 | SM d34:2 | C39 H77 N2 O6 P | 15.52 | [M+H]+ | 0.79 | 701.55865, 184.07330 | 95.5 |
| 150 | Spermidine | C7 H19 N3 | 0.53 | [M+H]+ | 0.45 | 146.16524, 129.13878, 72.08154 | 83.5 |
| 151 | Spermine | C10 H26 N4 | 0.53 | [M+H]+ | 0.21 | 203.22298, 112.11247, 84.08147 | 89.0 |

| | | | | | | | | |
|---|---|---|---|---|---|---|---|---|
| | 152 | Stearoyl ethanolamide | C20 H41 N O2 | 15.48 | [M+H]+ | 0.32 | 328.32111, 95.08614, 62.06094 | 92.2 |
| | 153 | Thiamine | C12 H16 N4 O S | 3.09 | [M+H]+ | 2.19 | 265.11234, 144.04808, 122.07155 | 79.4 |
| | 154 | 4-Hydroxy-proline | C5 H9 N O3 | 1.44 | [M+H]+ | 1.90 | 132.06577, 114.05529, 86.06069 | 76.1 |
| | 155 | Uracil | C4 H4 N2 O2 | 0.90 | [M+H]+ | 3.68 | 113.03497, 96.00880, 70.02959 | 88.9 |
| | 156 | Urocanic acid | C6 H6 N2 O2 | 0.76 | [M+H]+ | 0.55 | 139.05028, 121.03991, 93.04530 | 77.3 |
| | 157 | Valine | C5 H11 N O2 | 0.84 | [M+H]+ | 2.92 | 118.08694, 72.08155, 55.05516 | 82.7 |

Abbreviations: ESI, electrospray ionization; RT, retention time; LPC, lysophosphatidylcholine; LPE, lysophosphatidylethanolamine; LPG, lysophosphatidylglycerol; ACar, acetylcarnitine; PC, phosphatidylcholine; PE, phosphatidylethanolamine; SM, sphingomyelin.

**Supplementary Table S6.** Compounds identification in cell lipidomics in both ESI modes.

| ESI | No. | Name | Formula | RT (min) | Adduct | ppm | Fragments | Score |
|---|---|---|---|---|---|---|---|---|
| Neg | 1 | Linolenic Acid | C18 H30 O2 | 5.17 | [M-H]- | 4.59 | 277.21603 | 82.6 |
| | 2 | 12-Hydroxydodecanoic acid | C12 H24 O3 | 1.17 | [M-H]- | 2.78 | 215.16467 | 70.0 |
| | 3 | 16-Hydroxyhexadecanoic acid | C16 H32 O3 | 2.65 | [M-H]- | 0.44 | 269.21091, 253.21619, 225.22179 | 95.1 |
| | 4 | 6-Hydroxycaproic acid | C6 H12 O3 | 0.87 | [M-H]- | 8.99 | 131.07019, 111.04424, 85.06432 | 72.2 |
| | 5 | 8,11,14-Eicosatrienoic acid | C20 H34 O2 | 6.87 | [M-H]- | 0.21 | 305.24854, 261.25632 | 95.1 |
| | 6 | Arachidonic acid | C20 H32 O2 | 5.88 | [M-H]- | 0.44 | 303.23282, 259.24298 | 91.1 |

| | | | | | | | |
|---|---|---|---|---|---|---|---|
| 7 | Cer-NP t34:0 | C34 H69 N O4 | 11.35 | [M+COOH]- | 5.07 | 600.51782, 554.51508, 310.27481 | 70.0 |
| 8 | Chenodeoxycholic Acid | C24 H40 O4 | 2.43 | [M-H]- | 0.49 | 391.28519, 112.98399, 68.99428 | 88.7 |
| 9 | Deoxycholic acid | C24 H40 O4 | 2.65 | [M-H]- | 0.49 | 391.28519, 365.30597, 327.27014 | 91.1 |
| 10 | Hexadecanedioic acid | C16 H30 O4 | 1.40 | [M-H]- | 1.10 | 285.20682, 267.19620, 221.19002 | 73.1 |
| 11 | Lignoceric acid | C24 H48 O2 | 10.44 | [M-H]- | 0.83 | 367.35785 | 91.6 |
| 12 | LPE 16:0 | C21 H44 N O7 P | 3.97 | [M-H]- | 0.63 | 452.27798, 255.23257, 196.03719 | 71.2 |
| 13 | LPE 16:1 | C21 H42 N O7 P | 2.86 | [M-H]- | 0.93 | 450.26303, 253.21704, 196.03729 | 70.9 |
| 14 | LPE 18:0 | C23 H48 N O7 P | 5.45 | [M-H]- | 0.36 | 480.20939, 283.26404, 196.03723 | 72.2 |
| 15 | LPE 18:1 | C23 H46 N O7 P | 4.28 | [M-H]- | 0.70 | 478.29358, 281.24835, 196.03697 | 74.9 |
| 16 | LPE 20:1 | C25 H50 N O7 P | 5.62 | [M-H]- | 0.03 | 506.32523, 309.27982, 196.03708 | 73.5 |
| 17 | LPE 20:4 | C25 H44 N O7 P | 3.16 | [M-H]- | 0.07 | 500.27823, 303.23282, 196.03696 | 70.5 |
| 18 | LPG 18:1 | C24 H47 O9 P | 3.48 | [M-H]- | 1.22 | 509.28787, 281.24835, 152.99503 | 71.1 |
| 19 | Myristic acid | C14 H28 O2 | 5.40 | [M-H]- | 2.88 | 227.20100 | 78.5 |
| 20 | Oleic acid | C18 H34 O2 | 7.13 | [M-H]- | 0.80 | 281.24838 | 92.0 |
| 21 | Palmitic acid | C16 H32 O2 | 6.88 | [M+NH4]+ | 1.26 | 255.23267 | 97.5 |
| 22 | PC 30:0e | C38 H78 N O7 P | 11.03 | [M+COOH]- | 1.01 | 736.55054, 676.52887, 227.20096 | 70.8 |
| 23 | PC 30:1 | C38 H74 N O8 P | 9.65 | [M+COOH]- | 0.19 | 748.51355, 688.49237, 253.21678 | 70.3 |

| | | | | | | | |
|---|---|---|---|---|---|---|---|
| 24 | PC 30:1e | C38 H76 N O7 P | 10.57 | [M+COOH]- | 0.30 | 734.53436, 674.51282, 225.18544 | 71.4 |
| 25 | PC 36:1 | C44 H86 N O8 P | 12.40 | [M+COOH]- | 0.38 | 832.60699, 772.58582, 281.24823 | 79.7 |
| 26 | PC 36:1e | C44 H88 N O7 P | 12.98 | [M+COOH]- | 0.61 | 818.62854, 758.60699, 281.24841 | 70.9 |
| 27 | PC 36:2e | C44 H86 N O7 P | 12.19 | [M+COOH]- | 0.49 | 816.61279, 756.59094, 281.24835 | 72.7 |
| 28 | PC 38:2 | C46 H88 N O8 P | 12.45 | [M+COOH]- | 0.82 | 858.62366, 798.60205, 281.24838 | 71.4 |
| 29 | PC 38:3e | C46 H88 N O7 P | 12.60 | [M+COOH]- | 0.28 | 842.62781, 782.60675, 305.24838 | 74.6 |
| 30 | PE 32:0e | C37 H76 N O7 P | 11.03 | [M-H]- | 0.57 | 676.52905, 466.33078, 227.20102 | 79.7 |
| 31 | PE 32:1e | C37 H74 N O7 P | 12.24 | [M-H]- | 0.34 | 674.51324, 436.28244, 255.23256 | 76.1 |
| 32 | PE 32:2e | C37 H72 N O7 P | 11.43 | [M-H]- | 0.29 | 672.49756, 436.28250, 253.21695 | 73.5 |
| 33 | PE 34:0e | C39 H80 N O7 P | 13.18 | [M-H]- | 0.73 | 704.55945, 466.32833, 255.23248 | 72.8 |
| 34 | PE 34:1e | C39 H78 N O7 P | 13.10 | [M-H]- | 0.44 | 702.54462, 464.31226, 255.23257 | 72.9 |
| 35 | PE 34:2e | C39 H76 N O7 P | 12.35 | [M-H]- | 0.04 | 700.52869, 436.28287, 281.24832 | 72.0 |
| 36 | PE 34:3e | C39 H74 N O7 P | 11.53 | [M-H]- | 0.45 | 698.51270, 462.29834, 253.21690 | 73.6 |
| 37 | PE 34:4e | C39 H72 N O7 P | 11.28 | [M-H]- | 0.37 | 696.49762, 436.28235, 277.21698 | 74.3 |
| 38 | PE 36:1e | C41 H82 N O7 P | 13.30 | [M-H]- | 0.27 | 730.57581, 466.32990, 281.24835 | 73.0 |
| 39 | PE 36:2e | C41 H80 N O7 P | 13.20 | [M-H]- | 0.54 | 728.56036, 464.31476, 281.24841 | 72.4 |
| 40 | PE 36:3e | C41 H78 N O7 P | 12.43 | [M-H]- | 0.59 | 726.54474, 462.29959, 281.24841 | 72.8 |

| | | | | | | | | |
|---|---|---|---|---|---|---|---|---|
| | 41 | PE 36:4e | C41 H76 N O7 P | 11.68 | [M-H]- | 0.20 | 724.52881, 438.29916, 303.23267 | 72.8 |
| | 42 | PE 36:5e | C41 H74 N O7 P | 11.55 | [M-H]- | 0.10 | 722.51924, 436.28256, 303.23257 | 70.9 |
| | 43 | PE 38:4e | C43 H80 N O7 P | 12.81 | [M-H]- | 0.20 | 752.55981, 464.31454, 305.24838 | 79.1 |
| | 44 | PE 38:5e | C43 H78 N O7 P | 12.48 | [M-H]- | 0.15 | 750.54443, 464.31372, 303.23267 | 72.2 |
| | 45 | PE 40:5e | C45 H82 N O7 P | 13.07 | [M-H]- | 0.01 | 778.57562, 464.31461, 331.26398 | 75.0 |
| | 46 | Stearic acid | C18 H36 O2 | 8.40 | [M-H]- | 0.97 | 283.26398 | 93.1 |
| Pos | 47 | 13-HOTrE | C18 H30 O3 | 1.76 | [M+H]+ | 3.70 | 195.22568, 277.21576, 95.08594 | 79.4 |
| | 48 | 2-Arachidonoyl glycerol | C23 H38 O4 | 6.02 | [M+H]+ | 8.08 | 379.28122, 153.09132, 109.10128 | 83.5 |
| | 49 | 2,4-dihydroxyheptadec-16-en-1-yl acetate | C19 H36 O4 | 4.64 | [M+Na]+ | 2.34 | 351.24976, 147.08066, 81.07049 | 88.7 |
| | 50 | ACar 18:0 | C25 H50 N O4 | 4.86 | [M]+ | 2.04 | 428.37311, 85.02900 | 76.0 |
| | 51 | ACar 18:1 | C25 H48 N O4 | 3.76 | [M]+ | 2.21 | 426.35739, 85.02903 | 88.0 |
| | 52 | DAG 36:2 | C39 H72 O5 | 13.72 | [M+NH4]+ | 0.78 | 638.57230, 603.53394, 339.28903 | 70.3 |
| | 53 | Dipalmitoylphosphatidylcholine | C40 H80 N O8 P | 11.35 | [M+H]+ | 1.79 | 734.56812, 184.07312, 86.09691 | 98.6 |
| | 54 | Hexadecanamide | C16 H33 N O | 5.40 | [M+H]+ | 1.33 | 256.26315, 102.09149, 74.06052 | 94.3 |
| | 55 | Carnitine | C7 H15 N O3 | 0.75 | [M+H]+ | 0.74 | 162.11235, 103.03930, 60.08160 | 80.5 |
| | 56 | LPE 18:2 | C23 H44 N O7 P | 3.04 | [M+H]+ | 1.27 | 478.29221, 338.27328 | 74.2 |
| | 57 | N,N-Dimethylsphingosine | C20 H41 N O2 | 6.45 | [M+H]+ | 0.99 | 328.32068, 310.31070, 97.10143 | 78.4 |

| | | | | | | | |
|---|---|---|---|---|---|---|---|
| 58 | Oleamide | C18 H35 N O | 5.70 | [M+H]+ | 0.64 | 282.27896, 111.11721, 83.08613 | 97.1 |
| 59 | Oleoyl ethanolamide | C20 H39 N O2 | 5.40 | [M+H]+ | 1.21 | 326.30496, 247.24118, 62.06084 | 94.1 |
| 60 | Palmitoyl sphingomyelin | C39 H79 N2 O6 P | 10.38 | [M+H]+ | 0.98 | 703.57416, 184.07315, 86.09698 | 96.3 |
| 61 | Palmitoylcarnitine | C23 H45 N O4 | 3.46 | [M+H]+ | 1.66 | 400.34147, 144.10168, 85.02898 | 87.8 |
| 62 | PC 16:0e | C24 H50 N O7 P | 3.47 | [M+H]+ | 1.34 | 496.33914, 313.27188, 184.07320 | 84.4 |
| 63 | PC 16:1e | C24 H48 N O7 P | 2.48 | [M+H]+ | 0.96 | 494.32364, 311.25903, 184.07324 | 83.8 |
| 64 | PC 18:1e | C26 H52 N O7 P | 3.80 | [M+H]+ | 0.60 | 522.35510, 184.07321 | 85.9 |
| 65 | PC 20:1e | C28 H56 N O7 P | 5.13 | [M+H]+ | 1.32 | 550.38599, 184.07315 | 80.8 |
| 66 | PC 26:0 | C34 H68 N O8 P | 8.18 | [M+H]+ | 1.69 | 650.47443, 184.07306 | 74.2 |
| 67 | PC 28:0 | C36 H72 N O8 P | 9.30 | [M+H]+ | 1.98 | 678.50549, 450.29672, 184.07307 | 75.1 |
| 68 | PC 28:0e | C36 H74 N O7 P | 10.02 | [M+H]+ | 1.26 | 664.52673, 184.07320 | 88.7 |
| 69 | PC 28:1e | C36 H72 N O7 P | 9.89 | [M+H]+ | 0.39 | 662.51166, 184.07329 | 86.2 |
| 70 | PC 29:0e | C37 H76 N O7 P | 10.51 | [M+H]+ | 1.09 | 678.54248, 184.07318 | 88.7 |
| 71 | PC 29:1e | C37 H74 N O7 P | 10.24 | [M+H]+ | 1.50 | 676.52655, 184.07317 | 88.6 |
| 72 | PC 30:2e | C38 H74 N O7 P | 10.10 | [M+H]+ | 0.47 | 688.52789, 184.07317 | 92.3 |
| 73 | PC 31:0e | C39 H80 N O7 P | 11.36 | [M+H]+ | 2.32 | 706.57288, 184.07320 | 88.9 |
| 74 | PC 32:0e | C40 H82 N O7 P | 11.98 | [M+H]+ | 1.30 | 720.58923, 184.07317 | 89.2 |

| | | | | | | | |
|---|---|---|---|---|---|---|---|
| 75 | PC 32:1e | C40 H80 N O7 P | 11.19 | [M+H]+ | 1.26 | 718.57361, 184.07318 | 89.4 |
| 76 | PC 32:2e | C40 H78 N O7 P | 11.20 | [M+H]+ | 2.67 | 730.57294, 184.07321 | 93.4 |
| 77 | PC 32:3e | C40 H76 N O7 P | 10.81 | [M+H]+ | 1.28 | 714.54230, 184.07318 | 90.1 |
| 78 | PC 32:4 | C40 H72 N O8 P | 8.49 | [M+H]+ | 1.86 | 726.50818, 184.07317 | 73.8 |
| 79 | PC 33:0e | C41 H84 N O7 P | 12.46 | [M+H]+ | 2.13 | 734.60425, 184.07306 | 88.0 |
| 80 | PC 33:1e | C41 H82 N O7 P | 11.67 | [M+H]+ | 1.44 | 732.58911, 184.07314 | 88.6 |
| 81 | PC 33:2e | C41 H80 N O7 P | 10.96 | [M+H]+ | 2.16 | 730.57294, 184.07321 | 93.4 |
| 82 | PC 33:4e | C41 H76 N O7 P | 9.94 | [M+H]+ | 5.21 | 726.53943, 543.43616, 184.07314 | 78.2 |
| 83 | PC 34:0 | C42 H84 N O8 P | 12.28 | [M+H]+ | 1.47 | 762.59961, 184.07315 | 77.4 |
| 84 | PC 34:0e | C42 H86 N O7 P | 12.88 | [M+H]+ | 1.14 | 748.62061, 184.07323 | 89.7 |
| 85 | PC 34:1e | C42 H84 N O7 P | 12.78 | [M+H]+ | 1.78 | 746.60449, 184.07314 | 92.8 |
| 86 | PC 34:2e | C42 H82 N O7 P | 11.28 | [M+H]+ | 1.58 | 744.58899, 184.07318 | 90.3 |
| 87 | PC 34:3e | C42 H80 N O7 P | 10.76 | [M+H]+ | 1.88 | 742.57312, 184.07315 | 87.9 |
| 88 | PC 34:4 | C42 H76 N O8 P | 9.64 | [M+H]+ | 1.43 | 754.53705, 184.07323 | 73.9 |
| 89 | PC 34:4e | C42 H78 N O7 P | 10.28 | [M+H]+ | 2.10 | 740.55731, 184.07318 | 86.6 |
| 90 | PC 35:0e | C43 H88 N O7 P | 13.14 | [M+H]+ | 1.32 | 762.63611, 184.07330 | 87.9 |
| 91 | PC 35:1e | C43 H86 N O7 P | 12.33 | [M+H]+ | 5.39 | 760.61737, 184.07303 | 93.2 |

| | | | | | | | |
|---|---|---|---|---|---|---|---|
| 92 | PC 35:2e | C43 H84 N O7 P | 11.75 | [M+H]+ | 1.10 | 758.60498, 184.07312 | 93.4 |
| 93 | PC 35:3e | C43 H82 N O7 P | 11.06 | [M+H]+ | 3.09 | 756.58783, 184.07321 | 93.4 |
| 94 | PC 35:5e | C43 H78 N O7 P | 10.16 | [M+H]+ | 0.85 | 752.55823, 184.07317 | 88.1 |
| 95 | PC 36:0 | C44 H88 N O8 P | 12.47 | [M+H]+ | 9.89 | 790.62421, 184.07329 | 72.9 |
| 96 | PC 36:0e | C44 H90 N O7 P | 13.69 | [M+H]+ | 2.35 | 776.65094, 184.07320 | 87.3 |
| 97 | PC 36:2 | C44 H84 N O8 P | 11.76 | [M+H]+ | 1.20 | 786.59979, 184.07323 | 73.7 |
| 98 | PC 36:2e | C44 H86 N O7 P | 12.19 | [M+H]+ | 2.86 | 772.61926, 184.07303 | 92.6 |
| 99 | PC 36:3e | C44 H84 N O7 P | 12.18 | [M+H]+ | 3.38 | 770.60321, 184.07303 | 93.2 |
| 100 | PC 36:4 | C44 H80 N O8 P | 10.43 | [M+H]+ | 1.37 | 782.56836, 184.07315 | 73.5 |
| 101 | PC 36:4e | C44 H82 N O7 P | 11.07 | [M+H]+ | 1.45 | 768.58905, 184.07318 | 90.1 |
| 102 | PC 36:6 | C44 H76 N O8 P | 9.12 | [M+H]+ | 3.43 | 778.53546, 184.07314 | 73.4 |
| 103 | PC 37:0e | C45 H92 N O7 P | 14.08 | [M+H]+ | 1.49 | 790.66724, 184.07318 | 81.1 |
| 104 | PC 37:1e | C45 H90 N O7 P | 13.37 | [M+H]+ | 3.17 | 788.65527, 184.07312 | 89.6 |
| 105 | PC 37:2e | C45 H88 N O7 P | 12.63 | [M+H]+ | 1.83 | 786.63568, 184.07318 | 90.0 |
| 106 | PC 37:3e | C45 H86 N O7 P | 11.96 | [M+H]+ | 1.09 | 784.62061, 184.07327 | 91.9 |
| 107 | PC 37:4e | C45 H84 N O7 P | 11.29 | [M+H]+ | 1.07 | 782.60498, 184.07320 | 93.3 |
| 108 | PC 38:0e | C46 H94 N O7 P | 14.45 | [M+H]+ | 1.50 | 804.68286, 184.07320 | 87.8 |

| | | | | | | | |
|---|---|---|---|---|---|---|---|
| 109 | PC 38:1e | C46 H92 N O7 P | 13.69 | [M+H]+ | 2.78 | 802.67065, 184.07315 | 88.4 |
| 110 | PC 38:2e | C46 H90 N O7 P | 13.66 | [M+H]+ | 2.82 | 800.65051, 184.07298 | 92.0 |
| 111 | PC 38:4 | C46 H84 N O8 P | 11.67 | [M+H]+ | 2.14 | 810.59900, 184.07309 | 77.2 |
| 112 | PC 38:4e | C46 H86 N O7 P | 12.49 | [M+H]+ | 2.46 | 796.61951, 184.07320 | 93.1 |
| 113 | PC 38:5e | C46 H84 N O7 P | 11.37 | [M+H]+ | 2.63 | 794.20791, 184.07320 | 89.2 |
| 114 | PC 38:6e | C46 H82 N O7 P | 10.78 | [M+H]+ | 0.90 | 792.59088, 184.07318 | 89.3 |
| 115 | PC 38:7e | C46 H80 N O7 P | 10.94 | [M+H]+ | 2.54 | 790.57251, 184.07318 | 90.3 |
| 116 | PC 39:0e | C47 H96 N O7 P | 14.83 | [M+H]+ | 2.17 | 818.69794, 184.07314 | 81.2 |
| 117 | PC 39:2e | C47 H92 N O7 P | 13.43 | [M+H]+ | 1.99 | 814.67004, 184.07303 | 92.5 |
| 118 | PC 39:4e | C47 H88 N O7 P | 12.26 | [M+H]+ | 0.95 | 810.63635, 184.07317 | 85.6 |
| 119 | PC 39:5 | C47 H86 N O7 P | 11.82 | [M+H]+ | 1.06 | 808.62061, 625.51721, 184.07324 | 87.6 |
| 120 | PC 39:6e | C47 H84 N O7 P | 11.56 | [M+H]+ | 1.50 | 806.60461, 625.51318, 184.07317 | 81.7 |
| 121 | PC 40:0 | C48 H96 N O8 P | 14.67 | [M+H]+ | 2.01 | 846.69293, 184.07315 | 72.6 |
| 122 | PC 40:1 | C48 H96 N O7 P | 14.42 | [M+H]+ | 0.31 | 830.69946, 184.07320 | 89.5 |
| 123 | PC 40:2 | C48 H92 N O8 P | 13.25 | [M+H]+ | 1.82 | 842.66180, 184.07323 | 73.0 |
| 124 | PC 40:2e | C48 H94 N O7 P | 14.09 | [M+H]+ | 0.65 | 828.68353, 184.07327 | 88.6 |
| 125 | PC 40:3e | C48 H92 N O7 P | 13.45 | [M+H]+ | 0.99 | 826.66760, 184.07318 | 88.7 |

| | | | | | | | |
|---|---|---|---|---|---|---|---|
| 126 | PC 40:4 | C48 H88 N O8 P | 12.58 | [M+H]+ | 1.25 | 838.63098, 184.07318 | 75.1 |
| 127 | PC 40:4e | C48 H90 N O7 P | 12.74 | [M+H]+ | 1.62 | 824.65143, 184.07321 | 89.4 |
| 128 | PC 40:5e | C48 H88 N O7 P | 12.63 | [M+H]+ | 1.67 | 822.63574, 184.07321 | 90.9 |
| 129 | PC 40:6 | C48 H84 N O8 P | 11.43 | [M+H]+ | 2.00 | 834.59906, 184.07312 | 73.8 |
| 130 | PC 40:6e | C48 H86 N O7 P | 12.04 | [M+H]+ | 1.20 | 820.62048, 184.07321 | 88.8 |
| 131 | PC 40:7e | C48 H84 N O7 P | 11.90 | [M+H]+ | 2.22 | 818.60400, 184.07309 | 88.7 |
| 132 | PC 40:8 | C48 H80 N O8 P | 9.68 | [M+H]+ | 1.58 | 830.56812, 184.07320 | 72.7 |
| 133 | PC 40:8e | C48 H82 N O7 P | 10.25 | [M+H]+ | 3.64 | 816.59314, 184.07321 | 87.8 |
| 134 | PC 41:2e | C49 H96 N O7 P | 14.16 | [M+H]+ | 2.38 | 842.70172, 184.07314 | 89.7 |
| 135 | PC 41:3e | C49 H94 N O7 P | 13.61 | [M+H]+ | 1.11 | 840.68500, 184.07323 | 75.0 |
| 136 | PC 42:1e | C50 H100 N O7 P | 15.29 | [M+H]+ | 1.71 | 858.72955, 184.07317 | 88.1 |
| 137 | PC 42:2 | C50 H96 N O8 P | 14.26 | [M+H]+ | 1.12 | 870.69366, 184.07320 | 72.2 |
| 138 | PC 42:2e | C50 H98 N O7 P | 14.51 | [M+H]+ | 1.33 | 856.71423, 184.07318 | 88.4 |
| 139 | PC 42:3e | C50 H96 N O7 P | 14.42 | [M+H]+ | 2.58 | 854.69751, 184.07320 | 89.2 |
| 140 | PC 42:4 | C50 H92 N O8 P | 12.90 | [M+H]+ | 1.77 | 866.66180, 184.07314 | 71.2 |
| 141 | PC 42:4e | C50 H94 N O7 P | 13.95 | [M+H]+ | 2.34 | 852.68207, 184.07312 | 93.2 |
| 142 | PC 42:5e | C50 H92 N O7 P | 13.47 | [M+H]+ | 2.61 | 850.66620, 184.07314 | 88.9 |

| | | | | | | | |
|---|---|---|---|---|---|---|---|
| 143 | PC 42:6e | C50 H90 N O7 P | 12.33 | [M+H]+ | 1.00 | 848.65192, 184.07326 | 88.5 |
| 144 | PC 43:2e | C51 H100 N O7 P | 14.86 | [M+H]+ | 2.59 | 870.72876, 184.07318 | 89.9 |
| 145 | PC 44:2e | C52 H102 N O7 P | 15.23 | [M+H]+ | 3.28 | 884.74377, 184.07315 | 81.6 |
| 146 | PC 44:3e | C52 H100 N O7 P | 14.66 | [M+H]+ | 2.49 | 882.72882, 184.07320 | 85.4 |
| 147 | PC 44:4 | C52 H96 N O8 P | 14.00 | [M+H]+ | 2.38 | 894.69250, 184.07315 | 71.4 |
| 148 | PC 44:4e | C52 H98 N O7 P | 14.18 | [M+H]+ | 1.14 | 880.71436, 184.07315 | 77.8 |
| 149 | PC 44:5e | C52 H96 N O7 P | 13.92 | [M+H]+ | 1.40 | 878.69849, 184.07323 | 88.6 |
| 150 | PC 44:6e | C52 H94 N O7 P | 13.43 | [M+H]+ | 1.58 | 876.68268, 184.07317 | 87.6 |
| 151 | PC 46:5e | C54 H100 N O7 P | 14.71 | [M+H]+ | 2.16 | 906.72906, 184.07315 | 85.9 |
| 152 | PC 46:6e | C54 H98 N O7 P | 13.93 | [M+H]+ | 1.79 | 904.71375, 184.07323 | 87.7 |
| 153 | PC 46:7e | C54 H96 N O7 P | 13.29 | [M+H]+ | 1.29 | 902.69855, 184.07321 | 88.0 |
| 154 | PE 34:2 | C39 H74 N O8 P | 11.01 | [M+H]+ | 1.07 | 716.52325, 575.50287 | 89.2 |
| 155 | PE 38:2 | C43 H82 N O8 P | 12.75 | [M+H]+ | 1.34 | 772.58612, 631.56512 | 73.3 |
| 156 | SM d32:0 | C37 H77 N2 O6 P | 9.72 | [M+H]+ | 1.26 | 677.55835, 184.07320 | 90.7 |
| 157 | SM d32:1 | C37 H75 N2 O6 P | 9.29 | [M+H]+ | 1.26 | 675.54272, 184.07315 | 89.1 |
| 158 | SM d33:1 | C38 H77 N2 O6 P | 9.85 | [M+H]+ | 1.23 | 689.55835, 671.54834, 184.07320 | 90.3 |
| 159 | SM d34:0 | C39 H81 N2 O6 P | 10.78 | [M+H]+ | 1.62 | 705.58936, 184.07320 | 91.8 |

| | | | | | | | |
|---|---|---|---|---|---|---|---|
| 160 | SM d34:2 | C39 H77 N2 O6 P | 9.48 | [M+H]+ | 0.35 | 701.55945, 184.07320 | 86.3 |
| 161 | SM d35:1 | C40 H81 N2 O6 P | 10.71 | [M+H]+ | 8.99 | 717.58405, 184.07318 | 90.0 |
| 162 | SM d36:2 | C41 H81 N2 O6 P | 10.59 | [M+H]+ | 8.51 | 729.58429, 184.07315 | 90.4 |
| 163 | SM d38:1 | C43 H87 N2 O6 P | 12.37 | [M+H]+ | 0.65 | 759.63696, 184.07321 | 79.4 |
| 164 | SM d40:0 | C45 H93 N2 O6 P | 13.54 | [M+H]+ | 5.74 | 789.67987, 184.07307 | 90.3 |
| 165 | SM d40:1 | C45 H91 N2 O6 P | 13.24 | [M+H]+ | 1.92 | 787.66724, 184.07315 | 91.4 |
| 166 | SM d40:2 | C45 H89 N2 O6 P | 12.55 | [M+H]+ | 0.50 | 785.65271, 184.07326 | 88.7 |
| 167 | SM d42:0 | C47 H97 N2 O6 P | 14.30 | [M+H]+ | 5.53 | 817.71118, 184.07309 | 90.8 |
| 168 | SM d42:1 | C47 H95 N2 O6 P | 13.51 | [M+H]+ | 1.99 | 815.69843, 184.073223 | 91.3 |
| 169 | SM d42:2 | C47 H93 N2 O6 P | 13.21 | [M+H]+ | 1.89 | 813.68286, 795.67047, 184.07310 | 92.1 |
| 170 | SM d42:3 | C47 H91 N2 O6 P | 12.54 | [M+H]+ | 1.18 | 811.66779, 793.66345, 184.07301 | 78.8 |
| 171 | SM d43:1 | C48 H97 N2 O6 P | 14.25 | [M+H]+ | 3.02 | 829.71320, 184.07304 | 85.0 |
| 172 | SM d43:4 | C48 H91 N2 O6 P | 14.00 | [M+H]+ | 8.24 | 822.67554, 184.07323 | 89.6 |
| 173 | SM d44:1 | C49 H99 N2 O6 P | 14.74 | [M+H]+ | 1.48 | 843.73010, 184.07323 | 91.2 |
| 174 | SM d44:2 | C49 H97 N2 O6 P | 13.97 | [M+H]+ | 0.66 | 841.71515, 823.68243, 184.07327 | 90.3 |
| 175 | Sphingosine | C18 H37 N O2 | 2.81 | [M+H]+ | 4.04 | 300.32529, 282.27911, 121.10188 | 82.7 |

Abbreviations: ESI, electrospray ionization; RT, retention time; Cer, cermides; LPE, lysophosphatidylethanolamine; LPG, lysophosphatidylglycerol; ACar,

acetylcarnitine; DAG, diacylglycerol; PC, phosphatidylcholine; PE, phosphatidylethanolamine; SM, sphingomyelin.

**Supplementary Table S7.** ROC analysis of common differential metabolites in four different stages of CRC cells.

| ESI | Name | AUC (A) | AUC (B) | AUC (C) | AUC (D) |
|---|---|---|---|---|---|
| Neg | Xanthine | 1.00 | 1.00 | 1.00 | 1.00 |
| | Linolenic acid | 0.84 | 0.76 | 1.00 | 0.80 |
| | Arachidonic acid | 1.00 | 1.00 | 1.00 | 1.00 |
| | LPC 16:0 | 0.60 | 0.72 | 1.00 | 1.00 |
| | LPG 14:0 | 1.00 | 1.00 | 1.00 | 1.00 |
| | LPG 16:1 | 1.00 | 0.80 | 1.00 | 1.00 |
| | LPG 18:1 | 1.00 | 1.00 | 1.00 | 1.00 |
| | UDP-N-acetylglucosamine | 1.00 | 1.00 | 1.00 | 0.84 |
| | Methylmalonic acid | 1.00 | 0.80 | 1.00 | 1.00 |
| | Chenodeoxycholic acid | 1.00 | 1.00 | 1.00 | 1.00 |
| | Glycochenodeoxycholic acid | 1.00 | 1.00 | 0.96 | 0.52 |
| | Taurochenodeoxycholic acid | 1.00 | 1.00 | 1.00 | 0.64 |
| Pos | Betaine | 1.00 | 1.00 | 1.00 | 1.00 |
| | Proline | 1.00 | 1.00 | 1.00 | 1.00 |
| | Homoserine | 0.60 | 1.00 | 1.00 | 1.00 |
| | Tryptophan | 1.00 | 1.00 | 0.80 | 1.00 |
| | Isoleucine | 1.00 | 1.00 | 1.00 | 1.00 |
| | Norleucine | 0.96 | 1.00 | 0.96 | 0.96 |
| | Tyrosine | 1.00 | 0.84 | 1.00 | 1.00 |
| | 4-Hydroxy-proline | 1.00 | 1.00 | 1.00 | 1.00 |
| | Valine | 1.00 | 1.00 | 0.92 | 0.92 |
| | ACar 14:0 | 1.00 | 1.00 | 0.84 | 1.00 |
| | ACar 16:1 | 1.00 | 1.00 | 0.84 | 1.00 |
| | ACar 20:1 | 1.00 | 1.00 | 1.00 | 1.00 |
| | ACar 20:2 | 0.92 | 1.00 | 0.76 | 1.00 |
| | ACar 4:0 | 1.00 | 1.00 | 1.00 | 1.00 |
| | Decanoylcarnitine | 1.00 | 1.00 | 1.00 | 0.80 |
| | Hexanoylcarnitine | 1.00 | 1.00 | 0.84 | 1.00 |
| | Palmitoylcarnitine | 1.00 | 1.00 | 0.64 | 1.00 |
| | LPE 16:0 | 1.00 | 1.00 | 1.00 | 1.00 |
| | LPE 16:1 | 0.76 | 1.00 | 1.00 | 0.84 |
| | LPE 22:5 | 1.00 | 1.00 | 1.00 | 1.00 |
| | 5'-S-Methyl-5'-thioadenosine | 1.00 | 1.00 | 0.80 | 0.76 |
| | Adenine | 1.00 | 1.00 | 1.00 | 1.00 |
| | Guanine | 1.00 | 1.00 | 1.00 | 1.00 |
| | Nicotinamide adenine dinucleotide | 1.00 | 1.00 | 0.80 | 0.88 |
| | Oleoyl ethanolamide | 1.00 | 0.96 | 1.00 | 0.60 |
| | PC 20:1e | 0.80 | 1.00 | 1.00 | 1.00 |
| | PC 22:4e | 1.00 | 1.00 | 1.00 | 1.00 |
| | PC 22:5e | 1.00 | 1.00 | 1.00 | 1.00 |

| | | | | |
|---|---|---|---|---|
| Spermidine | 1.00 | 1.00 | 1.00 | 1.00 |
| Spermine | 1.00 | 1.00 | 1.00 | 1.00 |
| 17-Hydroxy-progesterone | 0.80 | 1.00 | 0.80 | 0.96 |
| Acetylcholine | 1.00 | 1.00 | 1.00 | 1.00 |
| Choline | 1.00 | 1.00 | 1.00 | 1.00 |
| N-Acetylneuraminic acid | 1.00 | 1.00 | 1.00 | 1.00 |

Abbreviations: ESI, electrospray ionization; RT, retention time; CRC, colorectal cancer; AUC, area under the curve; LPC, lysophosphatidylcholine; LPG, lysophosphatidylglycerol; ACar, acetylcarnitine; LPE, lysophosphatidylethanolamine; PC, phosphatidylcholine.

**Supplementary Table S8.** ROC analysis of common differential lipids in four different stages of CRC cells.

| ESI | Name | AUC (A) | AUC (B) | AUC (C) | AUC (D) |
|---|---|---|---|---|---|
| Neg | Deoxycholic acid | 1.00 | 1.00 | 1.00 | 1.00 |
| | LPE 16:0 | 1.00 | 1.00 | 1.00 | 1.00 |
| | LPE 16:1 | 0.56 | 1.00 | 1.00 | 1.00 |
| | LPE 18:0 | 1.00 | 1.00 | 1.00 | 1.00 |
| | LPE 18:1 | 1.00 | 1.00 | 1.00 | 0.92 |
| | LPE 20:1 | 1.00 | 1.00 | 1.00 | 1.00 |
| | LPE 20:4 | 0.80 | 1.00 | 0.52 | 0.60 |
| | PC 30:0e | 1.00 | 1.00 | 1.00 | 1.00 |
| | PC 30:1e | 0.80 | 1.00 | 0.96 | 1.00 |
| | PC 36:1e | 1.00 | 1.00 | 1.00 | 1.00 |
| | PC 38:2 | 0.96 | 1.00 | 1.00 | 1.00 |
| | PC 38:3e | 1.00 | 1.00 | 0.60 | 1.00 |
| | PE 32:0e | 1.00 | 1.00 | 0.64 | 1.00 |
| | PE 32:2e | 1.00 | 1.00 | 0.92 | 1.00 |
| | PE 34:2e | 1.00 | 1.00 | 1.00 | 1.00 |
| | PE 36:2e | 1.00 | 1.00 | 1.00 | 1.00 |
| | PE 36:4e | 1.00 | 1.00 | 1.00 | 1.00 |
| Pos | PC 16:1e | 0.60 | 0.56 | 0.52 | 0.60 |
| | PC 33:2e | 1.00 | 1.00 | 1.00 | 0.76 |
| | PC 35:1e | 1.00 | 0.96 | 0.80 | 1.00 |
| | PC 35:2e | 1.00 | 1.00 | 1.00 | 1.00 |
| | PC 35:3e | 1.00 | 1.00 | 1.00 | 1.00 |
| | PC 36:6 | 0.64 | 1.00 | 1.00 | 1.00 |
| | PC 37:3e | 1.00 | 1.00 | 1.00 | 0.52 |
| | PC 38:2e | 1.00 | 1.00 | 1.00 | 1.00 |
| | PC 40:2 | 0.56 | 1.00 | 0.56 | 0.80 |

| | | | | |
|---|---|---|---|---|
| PC 40:8 | 0.52 | 1.00 | 1.00 | 1.00 |
| SM d42:0 | 0.96 | 1.00 | 0.88 | 1.00 |
| Sphingosine | 1.00 | 1.00 | 1.00 | 1.00 |

Abbreviations: ESI, electrospray ionization; RT, retention time; CRC, colorectal cancer; AUC, area under the curve; LPE, lysophosphatidylethanolamine; PC, phosphatidylcholine; PE, phosphatidylethanolamine; SM, sphingomyelin.
